# Polychlorinated biphenyls modify *Arabidopsis* root exudation pattern to accommodate degrading bacteria, showing strain and functional trait specificity

**DOI:** 10.3389/fpls.2024.1429096

**Published:** 2024-07-05

**Authors:** Eleonora Rolli, Elisa Ghitti, Francesca Mapelli, Sara Borin

**Affiliations:** Department of Food, Environmental and Nutritional Sciences (DeFENS), University of Milan, Milan, Italy

**Keywords:** root exudates, plant-microbe interaction, rhizosphere, metabolomics, beneficial bacteria

## Abstract

**Introduction:**

The importance of plant rhizodeposition to sustain microbial growth and induce xenobiotic degradation in polluted environments is increasingly recognized.

**Methods:**

Here the “cry-for-help” hypothesis, consisting in root chemistry remodeling upon stress, was investigated in the presence of polychlorinated biphenyls (PCBs), highly recalcitrant and phytotoxic compounds, highlighting its role in reshaping the nutritional and signaling features of the root niche to accommodate PCB-degrading microorganisms.

**Results:**

*Arabidopsis* exposure to 70 µM PCB-18 triggered plant-detrimental effects, stress-related traits, and PCB-responsive gene expression, reproducing PCB phytotoxicity. The root exudates of plantlets exposed for 2 days to the pollutant were collected and characterized through untargeted metabolomics analysis by liquid chromatography–mass spectrometry. Principal component analysis disclosed a different root exudation fingerprint in PCB-18-exposed plants, potentially contributing to the “cry-for-help” event. To investigate this aspect, the five compounds identified in the exudate metabolomic analysis (i.e., scopoletin, N-hydroxyethyl-β-alanine, hypoxanthine, L-arginyl-L-valine, and L-seryl-L-phenylalanine) were assayed for their influence on the physiology and functionality of the PCB-degrading strains *Pseudomonas alcaliphila* JAB1, *Paraburkholderia xenovorans* LB400, and *Acinetobacter calcoaceticus* P320. Scopoletin, whose relative abundance decreased in PCB-18-stressed plant exudates, hampered the growth and proliferation of strains JAB1 and P320, presumably due to its antimicrobial activity, and reduced the beneficial effect of *Acinetobacter* P320, which showed a higher degree of growth promotion in the scopoletin-depleted mutant *f6’h1* compared to *Arabidopsis* WT plants exposed to PCB. Nevertheless, scopoletin induced the expression of the *bph* catabolic operon in strains JAB1 and LB400. The primary metabolites hypoxanthine, L-arginyl-L-valine, and L-seryl-L-phenylalanine, which increased in relative abundance upon PCB-18 stress, were preferentially used as nutrients and growth-stimulating factors by the three degrading strains and showed a variable ability to affect rhizocompetence traits like motility and biofilm formation.

**Discussion:**

These findings expand the knowledge on PCB-triggered “cry-for-help” and its role in steering the PCB-degrading microbiome to boost the holobiont fitness in polluted environments.

## 1 Introduction

By modulating their root chemistry, plants modify the composition of the exudation pattern to actively recruit beneficial microorganisms able to facilitate adaptation and protection from stresses (Afridi et al., 2024). Such event, referred to as “cry-for-help” (Rolfe et al., 2019), was originally documented as a plant response to herbivores and phytopathogen attacks (Yi et al., 2011; Berendsen et al., 2018), although mounting evidence suggests that it could have broader implications in plant functional requirements (Feng et al., 2023) and be deployed also under several abiotic stresses, like drought, salinity, and phosphate deficiency (Rizaludin et al., 2021). Moreover, modulation of the exudation pattern is considered a driving factor for the rhizoremediation of soil hydrocarbon pollution, providing nutrients to sustain the growth of hydrocarbon-degrading microorganisms and stimulating their catabolic activities (Rohrbacher and St-Arnaud, 2016). Therefore, it was proposed that the “cry-for-help” would represent an adaptative strategy exerted by the plant holobiont to grow in contaminated soils, and this process would become more relevant in the presence of poorly phyto-degradable, highly recalcitrant, and phytotoxic xenobiotics like polychlorinated biphenyls (PCBs) (Rolli et al., 2021).

PCBs constitute a class of 209 compounds, defined as congeners based on the number and position of the chlorine substituents. Although banned from production, they continue to pose a threat to humans, animals, and ecosystem health due to their recalcitrance, mutagenic and carcinogenic properties, and bioaccumulation in the food chain (Xiang et al., 2020). The biological degradation of PCBs is performed by a variety of microorganisms (Suman et al., 2021, 2024) and occurs through both anaerobic dechlorination, preferentially on highly chlorinated congeners (Xiang et al., 2020), and aerobic cleavage of the biphenyl ring, mainly on low-chlorinated congeners. These microbial functionalities are crucial for plant fitness since PCB pollution dramatically affects plant growth and development (Vergani et al., 2017a) by altering photosynthetic capacity and causing leaf chlorosis and by inducing an oxidative burst and the overload of the plant detoxification systems, which result to be inefficient due to the lack of specific enzymatic activities for PCB degradation (Lin et al., 2020). To counteract these detrimental effects, plants may recruit degrading bacterial populations from the soil microbiome in contaminated environments (Vergani et al., 2022) and sustain the establishment of a PCB-degrading microbiome in the rhizosphere, as observed for *M. sativa* L., *C. nigrescens* Willd., and *D. glomerata* L., plant species spontaneously growing in the historically polluted site Brescia-Caffaro in northern Italy (Vergani et al., 2017b). Specific secondary metabolites present in root exudation, like phenolics and terpenes, can enhance the bacterial degradative activity to catabolize PCBs and, in turn, alleviate the stress (Uhlik et al., 2013; Musilova et al., 2016).

Acknowledged as mediators between plants and the surrounding root microbiome, flavonoids are highly abundant phenolics in plant exudates with multifaceted capabilities to support plant fitness under stress (Ghitti et al., 2022). Due to their structural similarity with the PCB backbone, flavonoids were reported to act as co-metabolites and inducers for the expression of *bph*-encoded dioxygenases, responsible for the aerobic degradation of PCB congeners in *Pseudomonas putida* PML2 (Narasimhan et al., 2003) and *Rhodococcus erythropolis* U23A (Toussaint et al., 2012; Pham et al., 2015). In this context, we demonstrated that flavonoids play a wider and previously undescribed role in plant–microbe association in the presence of PCBs. We observed that flavonoid pure compounds can act as nutrients and signaling factors, differentially affecting bacterial growth, motility, chemotaxis, and early plant colonization in the PCB degrader strain *Paraburkholderia xenovorans* LB400: through these effects, flavonoids contributed to recruiting and sustaining the proliferation of strain LB400 in the plant rhizosphere (Ghitti et al., 2024). Nevertheless, in the long-term interaction between strain LB400 with *Arabidopsis* lines affected in flavonoid biosynthesis and exudation, it was hypothesized that other non-flavonoid metabolites could be responsible for LB400-triggered plant growth promotion and colonization under PCB stress (Ghitti et al., 2024).

Given these findings, this study aims to identify the PCB-induced fingerprint in root exudation and consequently determine the role of specialized metabolites, differentially produced under PCB stress, on the growth and activity of degrading bacteria. Despite metabolomics and exo-metabolomics representing key -omics approaches to investigate how root chemistry affects microbiome structure and functionality under adverse environmental conditions (van Dam and Bouwmeester, 2016), the framework of xenobiotic stress is still scarcely investigated. This limitation urges the exploration of PCB impact in plant exudation and its role in affecting the functionality of degrading microorganisms, with the vision to improve the knowledge on plant–microbe dynamics in contaminated environments and potentially optimize rhizoremediation strategies in terms, for instance, of selection of plant species with a selective rhizodeposition pattern.

Considering the sparse knowledge on the topic, this study sets the standards to disentangle the putative plant “cry-for-help” in the presence of PCBs. The approach is based on (i) the adoption of *Arabidopsis thaliana*, a valuable model plant to also investigate PCB-induced phytotoxicity (Zamcho et al., 2021; Ding et al., 2024; Bao et al., 2013; Jin et al., 2011), (ii) the trichlorobiphenyl congener PCB-18 as paradigm of this class of pollutants, considering that low-chlorinated congeners can be more easily up-taken by plant roots (Asai et al., 2002) and that this molecule was proved to exert detrimental effects on *Arabidopsis* growth (Bao et al., 2013) and other crops (Hao et al., 2020), and (iii) the versatile PCB-degrading strains *Acinetobacter calcoaceticus* P320 (Vergani et al., 2019), *Paraburkholderia xenovorans* LB400 (Chain et al., 2006), and *Pseudomonas alcaliphila* JAB1 (Zubrova et al., 2021). In this study, an *in vitro* assay has been developed to reproduce PCB phytotoxic hallmarks in *Arabidopsis* and used to analyze through liquid chromatography–mass spectrometry (LC–MS) the PCB-triggered modification in root chemistry exudation. Furthermore, the exudates identified in the metabolomic approach were further assayed to investigate their role in the growth, activity, and rhizocompetence traits (biofilm formation, motility) of three degradative bacteria. Overall, these findings contribute to unravel the PCB-stress-induced chemical remodeling of the rhizosphere niche that could favor the establishment of beneficial associations with bacteria able to catabolize this class of xenobiotics.

## 2 Materials and methods

### 2.1 Bacterial strains, plant material, culture media, and chemicals

The bacterial strains were routinely grown in tryptic soy broth (TSB, Merck, Darmstadt, Germany), Luria–Bertani (LB) broth, or Mineral Medium Brunner (DSMZ, Germany) supplemented with an adequate carbon source, either 30 mM sodium pyruvate or the other assayed metabolites, as indicated in the text. Bacterial cells were stored in glycerol stocks at -80°C and periodically revitalized on LB agar plates. *Arabidopsis thaliana* Col-0 WT and the *f6’h1* mutant, depleted in scopoletin biosynthesis (Stringlis et al., 2018), were used in the present study. PCB-18 (2,2′,5-trichlorobiphenyl, LCG Standards), solubilized in acetone, was used as the model PCB molecule. The root-exuded compounds identified by the metabolomic approach were hypoxanthine (Merck, Germany), solubilized in 1 N NaOH solution; scopoletin (Merck, Germany), solubilized in 80% methanol solution; N-(2-hydroxyethyl)-beta-alanine, (Merck, Germany); and the dipeptides L-seryl-L-phenylalanine (SF) and L-arginyl-L-valine (RV) (Twin Helix), solubilized in ultrapure MilliQ water.

### 2.2 Evaluation of *Arabidopsis thaliana* phytotoxicity to PCB-18 stress

This miniaturized assay allowed the evaluation of the phytotoxic effect exerted by increasing concentrations of PCB-18 on the fresh weight of *Arabidopsis* plantlets. *Arabidopsis thaliana* Col-0 seeds were surface-sterilized (Rolli et al., 2022) and spread on square petri dishes containing ½ Murashige and Skoog (MS) medium (2.2 g/L Murashige and Skoog basal medium, 0.5 g/L MES hydrate, pH = 5.4; 9 g/L agar type E). After vernalization (48 h, 4°C, in the dark), the plates were transferred in a growth cabinet (16-h light, 8-h darkness) at 22°C, 50% humidity, and 120–150 µmol/m light intensity. At 6 days after germination, three plantlets were transferred in the wells of a 24-well plate filled with 2 mL of MS medium containing 1% sucrose and supplemented with increasing concentrations of PCB-18. Three technical replicates were used for each treatment, and an equal volume of acetone was used for mock treatment; the experiment was repeated in two biological replicates. After 7 days of growth, the fresh weight of the plantlets was quantified using a high-sensibility scale and used to assess the phytotoxic effect induced by PCB-18 treatment compared to the mock treatment with acetone. The 70-µM concentration was selected to be used for further experiments (**Supplementary Figure S1**).

### 2.3 Root exudate collection assay from *Arabidopsis* plantlets exposed to PCB-18

A time-course assay was developed to collect root exudates (REs) and plant tissues and evaluate the PCB-induced phytotoxicity for the choice of the most appropriate time point to be analyzed via metabolomics. About 30 sterilized seeds were distributed in a 4.5-cm-diameter petri dish containing 10 mL of liquid ½ MS medium with 1% sucrose. After vernalization, the plates were transferred into the growth cabinet and grown for 11 days. The liquid nutrient medium was removed, and the plantlets were gently washed twice with 5 mL of ½ MS medium. After the washes, 10 mL of ½ MS medium was added to the petri dishes, containing either 70 µM PCB-18 to induce PCB stress or an equal volume of acetone as mock treatment. At this stage, sucrose was not added to the MS medium to prevent interference in the subsequent metabolomic analyses. The plants were then grown for 7 days. Four biological replicates were collected for PCB and mock treatments at the different time points of 2, 4, and 7 days (T2, T4, and T7) after PCB-18 treatments. For each replicate, the collected samples consisted of (i) the medium in which the plants were floating, which was considered as REs (Pantigoso et al., 2021) and was further subjected to metabolomic analysis and (ii) ~30 plantlets for RNA extraction and RT-qPCR quantification of the expression level of target PCB-responsive genes. The REs were filtered through a 0.45-μm filter (Millipore) to remove root debris, lyophilized, and stored at -80°C for further analyses. The plant material was rapidly frozen in liquid nitrogen and stored at -80°C for further analysis.

### 2.4 Evaluation of PCB-18 effect on plant growth

After 7 days of growth, the fresh weight of the plant shoots was quantified to verify the detrimental effect on plant growth exerted by the treatment with 70 µM PCB-18 compared to an equal volume of acetone as mock treatment. The plant fresh weight was measured by using a high-sensibility scale; number of plant shoots analyzed : 30, in three biological replicates.

### 2.5 Reactive oxygen species staining in *Arabidopsis* leaves

Reactive oxygen species (ROS) staining of *Arabidopsis* collected leaves was performed using 3,3′-diaminobenzidine (DAB) as described by Daudi and O’Brien (2012).

### 2.6 Ability of *Paraburkholderia xenovorans* LB400 to grow on *Arabidopsis* REs released under PCB-18 stress or under mock treatment

The collected REs from *Arabidopsis* Col-0 plants exposed to PCB stress or to mock treatment with acetone at the different time points (T2, T4, and T7) were used to assess their potential to sustain the growth of the model PCB degrader *P. xenovorans* LB400. To assess strain LB400 baseline growth, bacterial cells were resuspended in the *Arabidopsis* growth medium supplemented with PCB-18 or acetone, corresponding to ½ MS supplemented with 70 µM PCB-18 or acetone. This was the medium in which the plants were placed at the onset of the stress, as described in Section 2.3. This analysis was used to select the most appropriate time point of the plant root exudation profile to be further analyzed through the metabolomic approach. Strain LB400 was inoculated in 1/2 TSB liquid medium and incubated overnight at 30°C on a shaker (150 rpm). Subsequently, cells were collected and washed twice in physiological buffer by centrifugation (5 min, 4,000 rpm). Cells were then inoculated at a final concentration of 10^5^ cells/mL in triplicates in a 96-well plate using the collected REs as culture media. Negative controls without REs were set using 1/2 MS medium containing only 70 μM PCB-18 or an equal amount of acetone. The plate was incubated on a rotatory shaker at 30°C for 4 days. Bacterial cultures were then re-isolated and quantified by plating serial dilutions, obtaining the number of CFUs/mL.

### 2.7 RNA extraction from *Arabidopsis* seedlings and RT-qPCR to evaluate the relative expression of *Arabidopsis* genes that are responsive to PCBs, stress, and flavonoids

Seedlings were harvested at the indicated times (T2, T4, and T7 after PCB or mock treatments) and frozen in liquid nitrogen. Total RNA extraction from *Arabidopsis* plantlets was performed using the NucleoSpin RNA kit (Macherey-Nagel), including DNase treatment, following the manufacturer’s recommendations. RNA quantification and quality were measured using NanoDrop (Thermo Scientific). The cDNA was synthesized from 1 μg total RNA using oligo(dT) and RevertAid First Strand cDNA Synthesis kit (Thermo Fisher Scientific, USA) in a total volume of 20 μL. For cDNA synthesis, RNA was heated at 65°C for 5 min, the reverse transcription (RT) mix was added, and the reaction proceeded at 42°C for 1 h. cDNA was then heated at 70°C for 5 min and stored at 4°C. qPCR reactions were performed in a 12-μL final volume with 1 μL RT reaction product, 250 nM final concentration of each primer pair, and SsoAdvanced Universal SYBR Green Supermix (Bio-Rad, USA). The following genes were analyzed and the primer sequences are indicated by the literature references: *ABCG40* (Wrzaczek et al., 2009), *UGT73D1* (Langlois-Meurinne et al., 2005), *Germin-like protein subfamily 1 member 15* (Subramanian et al., 2018), and *XTR8* and *CYP707A3* (Rai et al., 2016). The results of the investigated genes are presented as relative expression by normalizing to the expression of the housekeeping gene *UBQ5* (At3g62250). Based on literature data, *UGT73D1* and *Germin-like protein subfamily 1 member 15* are PCB-responsive genes that are upregulated in the presence of tetrachlorobiphenyl (Subramanian et al., 2018) and dichlorobiphenyl (Jin et al., 2011), respectively. *UGT73D1* is a UDP-glucosyl transferase that is poorly characterized, although its expression seems to be stress-responsive, and it has also been described to be upregulated upon *P. syringae* exposure (Rehman et al., 2018). *Germin-like protein subfamily 1 member 15* belongs to a large family of enzymes that are induced in response to both biotic and abiotic stresses and that seem to affect plant defense by the formation of an oxidative burst response (Li et al., 2016). *ABCG40* is a xenobiotic-responsive gene involved in *Arabidopsis* resistance to lead contamination (Fan et al., 2016). Since flavonoids can act as inducers of the PCB catabolic pathway in bacterial cells (Narasimhan et al., 2003), flavonoid marker genes were also monitored, including *TT8*, the transcription factor for flavonoid biosynthesis, and its target genes *XTR8*, encoding for a hydrolase enzyme, and *CYP707A3*, a hydrolase induced under abiotic stress, including dehydration (Rai et al., 2016). The upregulation of cell-wall-remodeling enzymes has been linked to the activation of the metabolism of xenobiotics in plant tissues (Zamcho et al., 2021). All reactions were performed in CFX Connect Real-Time PCR Detection System (Bio-Rad) as follows: 95°C for 30 s, 40 cycles at 95°C for 5 s, and 60°C for 20 s; a dissociation step was programmed to validate the PCR products. Results were analyzed using CFX Manager Software (Bio-Rad). Statistical analysis was performed by applying the Mann–Whitney test.

### 2.8 Metabolomics analysis of root exudate composition

Sample preparation was performed according to MetaSysX standard procedure, a modified protocol from Salem et al. (2016). The analysis was originally performed by both LC–MS and gas chromatography–mass spectrometry, but statistically relevant results were obtained only with the LC–MS approach. For the LC–MS measurements of hydrophilic analytes, the samples were measured with Waters ACQUITY Reversed Phase Ultra Performance Liquid Chromatography (RP-UPLC) coupled to a Thermo-Fisher Exactive mass spectrometer. C18 columns were used for the hydrophilic measurements. Chromatograms were recorded in full-scan MS mode (mass range, 100–1,500). All mass spectra were acquired in positive and negative ionization modes. To ensure the highest standards of measurements, MetaSysX used the number of quality controls in each measurement queue. The queue was composed of quality control for the performance of instruments and batch-to-batch variation of intensity, quality control to compensate retention time deviation, and quality control for inter-batch deviation. Quality controls to compensate a potential intensity deviation are useful when bigger experiments are measured: in this experimental setup, the system is stable over time. The use of four replicates ensured the proper performance of the experiment. For LC–MS data processing, data extraction was accomplished with the software PeakShaper (MetaSysX GmbH). The alignment and filtration of LC–MS data were completed using an in-house software. After extraction from the chromatograms, data were processed, aligned, and filtered for redundant peaks. The alignment of the extracted data from each chromatogram was performed according to the criterion that a feature had to be present in all replicates of at least one of the groups. At this stage, the average RT and m/z values are given to the features. The alignment was performed for each type of measurement independently. Data alignment was followed by the application of various filters to refine the dataset, which included the removal of (i) isotopic peaks, (ii) in-source fragments of the analytes (due to the ionization method), and (iii) redundant peaks like additional less intense adduct of the same analyte and redundant derivatives, to guarantee the quality of the data for further statistical analyses.

For the annotation, the in-house MetaSysX database of chemical compounds was used to match the features detected in the LC–MS polar platform. MetaSysX possesses in-house databases created internally, which contain the spectral and retention time information of 8,000 reference compounds available as pure compounds and measured in the same chromatographic and spectrometric conditions as the measured samples. The annotation of the content of the sample was performed by database query of mass-to-charge ratio and the retention time of detected features within certain criteria, corresponding to 6 ppm and 0.1 min of deviation from the reference compound mass-to-charge ratio and retention time, respectively, for the polar and non-polar platforms. Co-eluting compounds with the same mass-to-charge ratio were all kept. Only the matched compounds that passed the indicated criteria were kept. Normalization was performed for each platform separately for the median of intensities of each sample. Normalized intensities were merged as a final data matrix. Among the annotated metabolites, N-hydroxyethyl-β-alanine was identified, based on the MetaSysX database. For this compound, the alpha isomer was not present in the database, and it cannot be excluded, but the following experiments were performed using the beta isomer. For statistical analysis, all normalized intensities were log_2_-transformed. For heatmap visualization, the logarithmically transformed values were scaled by median-centering. The missing values were not replaced and color-coded with white. The test was conducted on the log_2_-transformed intensities. The statistical test was computed with a two-tailed *t*-test assuming unequal variance, and *p*-values were corrected using the Benjamini–Hochberg (BH) method. Principal component analysis (PCA) was performed on normalized intensities in PRIMER v. 6.1, PERMANOVA+ for PRIMER routine (Anderson et al., 2008). Significant differences in the metabolite fingerprints were investigated by permutational analysis of variance, PERMANOVA (Anderson et al., 2008), considering the factor “treatment” for mock (acetone) and PCB-18 (treated).

### 2.9 Generation of fluorescence-labeled strains for microscopy analysis of the root colonization pattern

Strain LB400 was previously labeled with the *mScarlet* by adopting a conjugation protocol that was applied also to successfully label *Pseudomonas* JAB1 (Ghitti et al., 2024). For the *gfp*-tagging of *Acinetobacter* P320, the protocol described in Rolli et al. (2015) was applied. The root colonization experiment was run according to Rolli et al. (2021). The microscopy analysis was performed at the platform Unitech NOLIMITS available at the University of Milan. The fluorescence emitted by the fluorescent-tagged bacteria colonizing *Arabidopsis* plantlets was observed with a stereomicroscope (Stereo Nikon SMZ) by scanning the root system at ×15 magnification. For an optimized visualization of the mScarlet-labeled strains on the *Arabidopsis* root system, the maximum brightness of the epifluorescence microscopy images was adjusted to value 150 by using ImageJ software. For the *gfp* signal, the maximum brightness of the epifluorescence microscopy images was adjusted to value 190 by using ImageJ software.

### 2.10 *In vitro* assay for the putative antimicrobial effect of scopoletin

The putative antimicrobial effect induced by the presence of scopoletin was tested and quantified *in vitro* by employing the microplate assay proposed by Stringlis et al. (2018) with modifications. In brief, 96-well plates (VWR, USA) were filled with 100 μL of TSB supplemented with different concentrations of scopoletin (0.25, 0.5, 1, and 2 mM). The negative control was composed of equal amounts of the solubilizing solvent (80% methanol), while antibiotic tetracycline (100 μg/mL, Merck, Germany) was used as the positive control for the inhibition of bacterial growth. The three bacterial strains were grown overnight in 1/2 TSB medium until late-log phase, and 2 mL of bacterial cultures was harvested, washed twice, and resuspended in physiological buffer. For the assay, 100 μL of the bacterial cell suspension was added to each well containing the TSB supplemented with scopoletin to reach a final concentration of 10^8^ cells/mL in the well. The growth was monitored by measuring OD_600_ every hour for 24 h using a 96-well plate reader (Tecan, Switzerland), keeping the plate incubated at 30°C and shaking for 7 s before each measurement. Each condition was tested with three biological replicates and three technical replicates, respectively. Relative growth to express bacterial sensitivity to scopoletin was calculated as reported by Harbort et al. (2020) by dividing the final OD_600_ measurement (at time 24 h) of each concentration assayed by the OD_600_ obtained in the root-exudate-free control. Doubling time and maximum growth rate were calculated as specified by Navarro-Pérez et al. (2022).

### 2.11 Evaluation of scopoletin on *Acinetobacter* P320–plant interaction under control conditions and in the presence of PCB-18 stress

*Acinetobacter* P320, the bacterial strain most affected by scopoletin, was evaluated for its performance in plant–bacteria interaction in wild-type (WT) *Arabidopsis* plants and in *f6’h1* mutant depleted in scopoletin biosynthesis (Stringlis et al., 2018). Sterilized seeds were sown on ½ MS agar plates (50 mL) supplemented with *Acinetobacter* P320 at a concentration of 2 × 10^5^ cells/mL or without the bacterial inoculum in the case of control seeds that were prepared by adding an equal volume of physiological buffer. The plates were vernalized for 2 days at 4°C in the dark and then placed vertically in a growth cabinet for 5 days (22°C, 50% humidity, long-day conditions with light intensity of 120–150 µmol/m). At 5 days after germination, the *Arabidopsis* plantlets were transferred onto fresh ½ MS plates containing 20 µM PCB-18 (treated) or an equal volume of acetone (mock treatment) as described in Ghitti et al. (2024). The plates were incubated for a total of 14 days in vertical position in a growth cabinet. At the end of the experiment, the fresh weight of the plantlets was measured by using a precision scale. All measurements were performed on three independent experiments and on at least seven plants per condition. The beneficial index, which means the growth promotion ability exerted by *Acinetobacter* P320, was calculated as the ratio between the fresh weight of *Acinetobacter* P320-colonized plants and non-inoculated seedlings and was expressed in percentage in the same genetic background. The beneficial index was used as a tool to compare *Acinetobacter* P320 improving performances under PCB-18 stress in the *Arabidopsis* backgrounds used in the present assay. To evaluate the bacterial colonization efficiency, the root systems were collected and placed in pre-weighed Eppendorf tubes, and their fresh weight was measured. The roots were then homogenized with TissueLyser II (QIAGEN, Germany) using the following protocol: two cycles at 20-Hz frequency for 20 s and, after adding 900 µL of physiological buffer, two cycles at 15 Hz for 1 min. The smashed root suspension obtained was used as 10^-1^ suspension to prepare serial dilutions for the drop-plate count method, which were plated on LB plates. After an overnight incubation at 30°C, the bacterial colonies were counted, and the root colonization efficiency was expressed as CFUs/mg root fresh weight.

### 2.12 Induction of *bphA* gene expression by scopoletin in *Pseudomonas* JAB1 and in *Paraburkholderia* LB400

Adapting the protocol used by Zubrova et al. (2021), *Pseudomonas* JAB1 cells were grown in MMB supplemented with 30 mM sodium pyruvate on a rotatory shaker at 30°C until late-log phase. The culture was then harvested by centrifugation, washed in physiological buffer, and resuspended in 1 OD/mL of fresh MMB supplemented with 30 mM sodium pyruvate. Aliquots of the bacterial suspension were divided into glass vials, previously amended with 0.25 mM scopoletin and biphenyl; the latter was used as the positive control for *bphA* induction. Media supplemented only with the solvents in which scopoletin and biphenyl were solubilized, 80% methanol and acetone, respectively, were used as the negative controls. The solvents were evaporated for 15 min prior to the addition of the bacterial suspension. The cultures were incubated at 30°C on a rotatory shaker, and for each condition, 200 µL of culture was sampled in triplicate at time points 0 and 2 h and then pelleted by centrifugation (4,000 rpm, 4°C, 5 min). The pellet obtained was stored at -20°C for subsequent RNA extraction steps. Total RNA extraction and RT-qPCR were performed as previously reported (Ghitti et al., 2024). Relative quantification was performed using JAB1 *bphA* as target gene (primers: F: 5′-GAGATCCAGAAGGGGCTAC-3′; R: 5′-GCGCATCCAGTGGTGATA-3′) and *infB* as reference gene (primers: F: 5′-AGTGACCGATAGTGAGAAAC-3′; R: 5′-AACACTGATGGTCTTGCTAC-3′). Data analysis to calculate the relative abundance of *bphA* gene expression was performed as reported previously (Ghitti et al., 2024).

The *bph* operon induction in *Paraburkholderia* LB400 with scopoletin was performed following the assay previously described in Ghitti et al. (2024).

### 2.13 *In vitro* growth on pure REs as unique carbon or nitrogen sources

The ability of the bacterial strains to use hypoxanthine, L-seryl-L-phenylalanine, and L-arginyl-L-valine, the plant REs that increased their abundance upon PCB-18 stress, as unique sources of carbon or nitrogen was tested *in vitro* in liquid culture. The bacterial strains were cultivated overnight in MMB medium supplemented with 30 mM sodium pyruvate at 30°C on a rotatory shaker (150 rpm), and cells were harvested by centrifugation (10 min, 4,000 rpm). Subsequently, bacterial cultures were washed twice in physiological buffer and resuspended at a final concentration of 5 × 10^5^ cells/mL in MMB supplemented with 10 mM of the pure compounds identified in the metabolomic approach as carbon sources. MMB with no carbon source was used as the negative control, while MMB supplemented with 10 mM sodium pyruvate was used as the positive control for bacterial growth. For growth on REs as unique nitrogen source, bacterial cultures were washed twice in physiological buffer and resuspended at a final concentration of 5 × 10^5^ cells/mL in MMB deprived of (NH_4_)_2_SO_4_ and supplemented with 4 mM REs as nitrogen source instead. MMB with no nitrogen source was used as the negative control, while the conventional composition of MMB was used as the positive control for bacterial growth. The cultures were aliquoted (200 µL per well) into a transparent 96-well plate (VWR, USA). Abiotic controls were analyzed as blanks to subtract the absorbance background given by the medium. Each condition was tested with three biological replicates and three technical replicates, respectively. Bacterial growth was monitored by measuring the optical density at 600 nm every hour for 48 h using a 96-well plate reader (Tecan, Switzerland), keeping the plate in incubation at 30°C and shaking for 7 seconds before each measurement.

### 2.14 *In vitro* biofilm formation assay

Biofilm formation was estimated *in vitro* by quantifying the bacterial cell adhesion to a solid surface (polystyrene 96-well plate, VWR, USA) using crystal violet (CV) staining, following the method applied by Yoshioka and Newell (2016) and previously adapted by Ghitti et al. (2024). For the assay, the pure compounds of the identified root exudates were added at the final concentrations of 5, 50, and 500 µM. The respective solvents were added to the medium as the negative controls, and non-inoculated medium was aliquoted as the blank. Each condition was tested with three independent biological replicates.

### 2.15 *In vitro* swimming motility assay

The effect of pure REs on bacterial swimming motility was tested for the strains JAB1 and LB400 in soft agar plates with 1/10 TSB medium supplemented with 0.25% (w/v) agar and 50 and 100 μM REs following the indications of Kearns (2010) and Bartolini and Grau (2019) and previously adapted by Ghitti et al. (2024). Each condition was tested in three biological replicates, each with three technical replicates.

### 2.16 Bacterial growth assay in the presence of increasing concentrations of pure root exudate compounds

For this *in vitro* growth assay, the protocol used by Huang et al. (2019) was applied with some adaptations as previously reported (Ghitti et al., 2024). Bacterial strains were grown in 1/10 TSB supplemented with pure compounds that were enriched in *Arabidopsis* REs secreted under PCB stress (hypoxanthine, L-seryl-L-phenylalanine, and L-arginyl-L-valine) at final concentrations of 10, 20, 50, and 100 µM. Each condition was tested with three biological replicates and three technical replicates, respectively. Bacterial growth was monitored by measuring the optical density at 600 nm every hour for 24 h using a 96-well plate reader (Tecan, Switzerland).

### 2.17 Statistical analysis

Statistical analyses were performed using R and Graphpad. Normal data were tested using ANOVA followed by unpaired *t*-test for multiple comparisons (95% confidence interval). For non-normal data, Kruskal–Wallis test was adopted, followed by Dunn’s *post-hoc* test (95% confidence interval). To compare non-normal distributions with small sample size (*n* < 30), Mann–Whitney non-parametric test was used.

## 3 Results

### 3.1 *In vitro* assay to detect the PCB-18-triggered “cry-for-help” signature in *Arabidopsis thaliana* and root exudation collection

**Figure 1:**
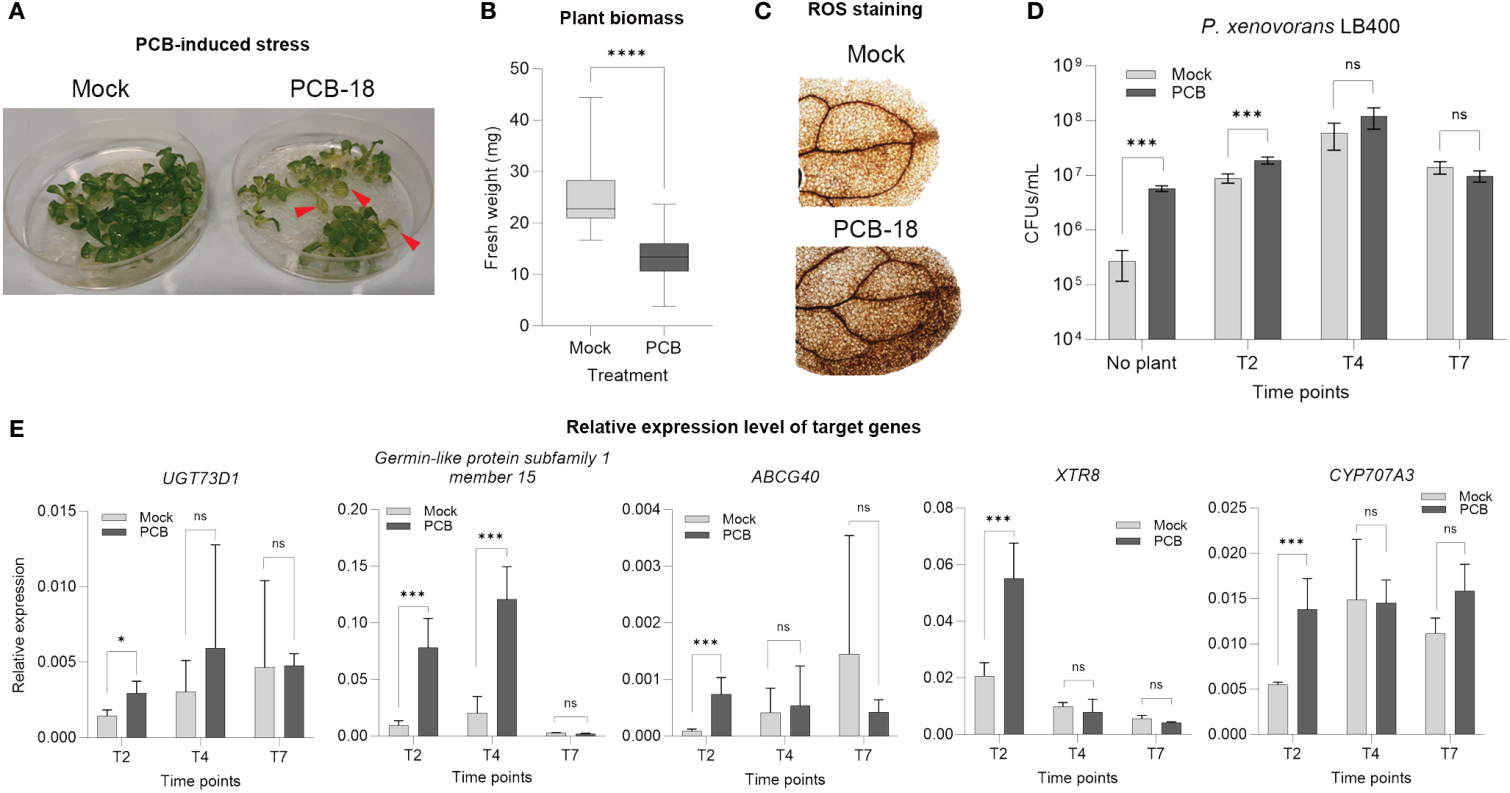
Validation of the *in vitro* assay to simulate polychlorinated biphenyl (PCB) stress in *Arabidopsis thaliana* and selection of the appropriate time point for the metabolomic analysis of root exudates. **(A)** Upon exposure for 7 days to 70 µM PCB-18, the *Arabidopsis* plantlets showed leaf chlorosis, as indicated by the red arrows. **(B)** PCB-18-induced stress showed a severe decrease in fresh biomass for plantlets at T7 treated with 70 µM PCB-18 compared to plants exposed to an equal volume of acetone (mock). Statistical analysis was performed by applying unpaired *t*-test. ****:*p* ≤ 0.0001, *n* = 30. **(C)** ROS staining on the leaves of plantlets at T7 exposed to PCB stress or mock treatment. **(D)** Ability of the PCB degrader strain *Paraburkholderia xenovorans* LB400 to exploit as nutrient source the root exudate-enriched medium released by *Arabidopsis* plantlets exposed to PCB-18 or mock treatment at different time points (T2, T4, and T7). The medium for the sample indicated as “no plant” corresponded to ½ MS supplemented with 70 µM PCB-18 (dark gray bar) or acetone (light gray bar). Statistical analysis was performed by applying the Mann–Whitney test (*n* = 3). ***:*p* ≤ 0.001; ns, non-statistically relevant. **(E)** RT-qPCR representing the relative expression level of PCB-, xenobiotics-, and flavonoid-responsive genes. The plantlets were collected at different time points upon PCB or mock treatment at T2, T4, and T7. Transcript accumulation is expressed as relative to the average of the transcript level of *UBQ5* as reference gene, according to Serra et al. (2013). Statistical analysis was performed by applying the Mann–Whitney test (3 < *n* > 4). *:*p* ≤ 0.05; ***:*p* ≤ 0.001; ns, non-statistically relevant.

Based on the reduction in *Arabidopsis* plantlet fresh biomass in presence of PCB-18 (**Supplementary Figure S1**), a miniaturized *in vitro* assay was developed to reproduce PCB phytotoxic features in *Arabidopsis thaliana*. At 7 days after 70 µM PCB-18 application, the stressed plants showed leaf chlorosis (**Figure 1A**), a 44% reduction in fresh biomass (**Figure 1B**), and enhanced ROS accumulation in the leaves (**Figure 1C**), indicating that the assay was effective in recapitulating the main hallmarks of PCB stress. Plantlets and RE-enriched media were analyzed at different time points (2, 4, and 7 days after PCB treatment) to select the most suitable one to collect REs from for metabolomics analysis.

The model PCB-degrader *Paraburkholderia xenovorans* LB400 demonstrated enhanced growth ability in the presence of the medium enriched with REs collected at T2, while no statistically relevant differences were observed for T4 and T7 time points by comparing the REs released by *Arabidopsis* plantlets in the presence of acetone or PCB-18 (**Figure 1D**). Furthermore, RT-qPCR analysis was applied to monitor the relative expression of PCB stress and flavonoid-responsive genes, knowing the interconnection between these plant secondary metabolites and the stimulation of microbial degraders (Ghitti et al., 2022). All the analyzed genes showed a statistically relevant increase in their expression level upon PCB-18 treatment at T2; in the case of the PCB-responsive genes *UGT73D1* and *Germin-like protein subfamily 1 member 15*, this increase was observed also at T4 in PCB-treated plantlets (**Figure 1E**). The T2 time point was selected for subsequent RE metabolomic analysis, potentially representing the root exudation fingerprint specifically triggered by PCB-18 and affecting microbial degraders.

### 3.2 Untargeted metabolomics analysis on PCB-18-triggered REs in *Arabidopsis*

**Figure 2:**
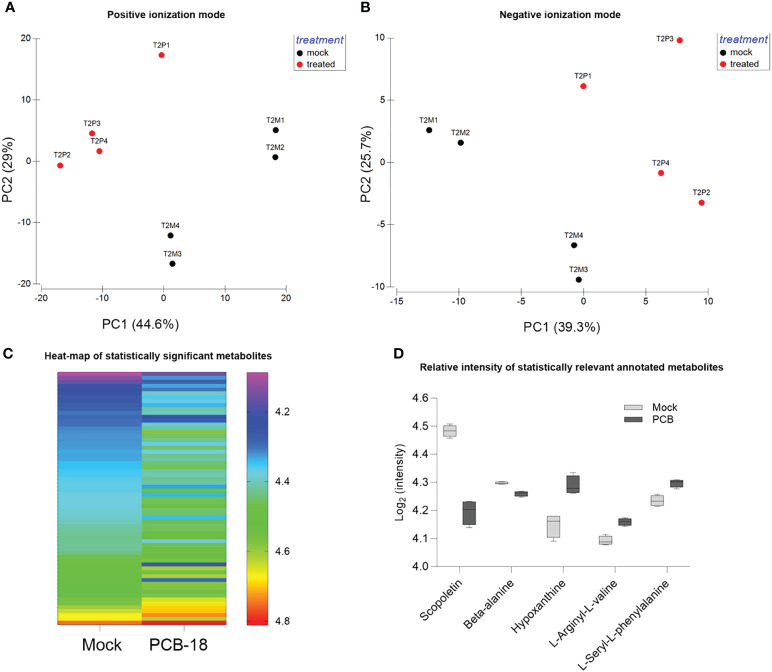
*Arabidopsis* root exudate compounds diverge in response to PCB-18. **(A, B)** Principal component analysis of the detected metabolite features corresponding to the rhizodeposition released by plantlets exposed for 2 days (T2) to 70 µM PCB-18 (T2P, in red in the graph) or mock-treated with an equal amount of acetone (T2M, in blue in the graph) as detected by LC–MS analysis in positive (1,650 features) and negative ionization (863 features) modes, respectively. **(C)** Heatmap of the 65 metabolic features that showed the differential relative abundancies among PCB-18-treated root exudates compared to the acetone-treated ones. The metabolites have a mean ratio fold change ≥1 and a *p*-value ≤0.05, corrected using the Benjamini–Hochberg (BH) method. In the acetone-treated sample, the metabolites were ordered from the one with the lowest relative intensity (top of the graph) to the one with the highest relative intensity (bottom of the graph). In the PCB-18 bar, the corresponding relative intensity for each metabolite is shown, according to the heat-color map. **(D)** Boxplot showing the relative intensity of the annotated metabolites, expressed as log_2_ of the normalized intensity: scopoletin, β-alanine, hypoxanthine, L-arginyl-L-valine, and L-seryl-L-phenylalanine in the root exudates released by *Arabidopsis* plantlets under PCB-18 stress or in the mock treatment.

The metabolomic analysis detected 2,513 metabolite features comprising 200 annotated ones (**Supplementary Table S1**; **Supplementary Figures S2, S3**). PCA analysis of metabolite fingerprint was performed to identify similarities/dissimilarities among REs exuded with PCB-18 or with acetone as mock treatment in the medium (**Figures 2A, B**). The PCA plots (**Figures 2A, B**) indicated that, based on the metabolite profile, the samples clustered differently according to the treatment (i.e., PCB-exposed plantlets and mock-treated ones, respectively). Axis 1 and axis 2 of the PCA plots explain 73.6% and 65% of the total variance for the metabolite detected in positive and negative ionization mode in the LC–MS analysis, respectively (**Figures 2A, B**). As suggested by PCA, PERMANOVA analysis (**Supplementary Table S3**) confirmed that the samples differed according to the treatment for both metabolites detected in positive mode (PERMANOVA: *p* = 0.0286) and for those detected in negative mode (PERMANOVA: *p* = 0.0283).

In the metabolite dataset, 65 features showed a statistically different relative abundance between the REs of mock-treated and PCB-18-exposed plants (**Figure 2C**; **Supplementary Table S** **2**). These features may represent the PCB-18-induced “cry-for-help” in *Arabidopsis* exudation pattern, and the variation in their relative abundance among treatments is represented in the heatmap, with 10 features showing a relative increase under PCB-18 stress, while 55 features decreased their relative abundance (**Figure 2C**). Five of these compounds were identified and corresponded to scopoletin and N-hydroxyethyl-beta-alanine (β-alanine), which showed a decrease upon PCB-18 treatment, and hypoxanthine, L-seryl-L-phenylalanine (SF), and L-arginyl-L-valine (RV) that increased their relative abundance in PCB-exposed REs (**Figure 2D**). The only flavonoid compounds identified in the dataset are kaempferol, dihydro-kaempferol, and genistein 7-O-β-D-glucoside, but they did not show a significant change in their relative abundance in the PCB-18 treatment (**Supplementary Figure S4**). In the vision that the identified exudates are part of the “cry-for-help” strategy exerted by the host plant under PCB stress, these metabolites were further assayed, as pure compounds, to investigate their putative role on the physiology of three degrading bacteria that were able to colonize the *Arabidopsis* root system (**Supplementary Figure S5**) and that were previously isolated from the plant rhizosphere in polluted soils.

### 3.3 Scopoletin negatively influenced the growth of PCB-degrading bacteria

**Figure 3:**
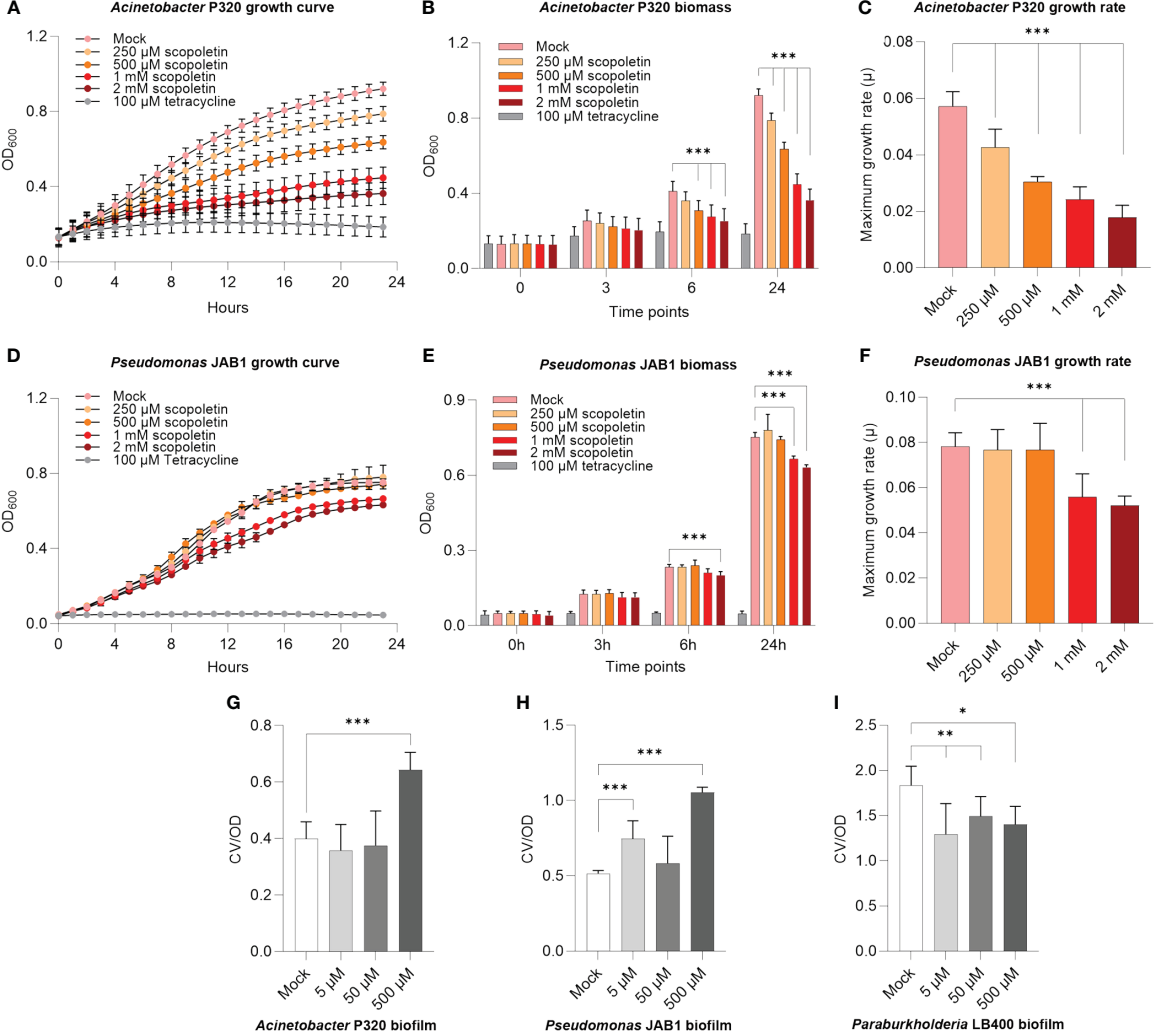
Scopoletin negatively affected the growth of *Acinetobacter* P320 and *Pseudomonas* JAB1. **(A, D)** Growth curves of *Acinetobacter* P320 and *Pseudomonas* JAB1, respectively, at increasing concentrations of scopoletin. In the assay, 100 µM tetracycline was used as the positive control for bacterial growth inhibition. **(B, E)** Bacterial biomass accumulation at different time points during the growth of *Acinetobacter* P320 and *Pseudomonas* JAB1, respectively, at increasing concentrations of scopoletin. ***:*p* ≤ 0.001. **(C, F)** Values of *Acinetobacter* P320 and *Pseudomonas* JAB1 maximum growth rate when exposed to increasing concentrations of scopoletin. For all the graphs, statistical analysis was performed using the Mann–Whitney test by comparing the scopoletin treatment with the mock solvent control. ***:*p* ≤ 0.001. **(G–I)** Influence of scopoletin on the biofilm formation ability of *Acinetobacter* P320, *Pseudomonas* JAB1, and *Paraburkholderia* LB400, respectively, represented as the ratio between the crystal violet OD (CV) of the stained biofilm and the optical density of the culture at 600 nm (OD). Statistical analysis was performed using the Mann–Whitney test (*n* = 3) by comparing the scopoletin treatment with the mock solvent control. *:*p* ≤ 0.05; **:*p* ≤ 0.01; ***:*p* ≤ 0.001.

Scopoletin is a well-described plant secondary metabolite that mediates host–microbe interactions (Stringlis et al., 2019; Voges et al., 2019). Since the relative abundance of this compound decreased in the REs of PCB-stressed plants (**Figure 2D**), we verified if it could negatively affect the growth of PCB-degrading bacteria, based on previous observations of scopoletin antimicrobial activity on beneficial/pathogenic microbes (Stringlis et al., 2018). *Acinetobacter* P320 was highly inhibited by scopoletin even at the lowest tested concentration of 250 µM (**Figure 3A**; **Supplementary Figure S6A**), showing a severe decrease in biomass accumulation already after 6 h upon scopoletin supplementation (**Figure 3B**) and an overall decrease of its relative growth after 24 h of exposure to this plant secondary metabolite (**Figure 3C**). Furthermore, 1 and 2 mM scopoletin negatively affected the doubling time of the bacterium (**Supplementary Table S4**), also causing a concentration-dependent reduction of the maximum growth rate compared to mock treatment (percentage of reduction: 25.5% at 500 µM and 56% at 2 mM) (**Figure 3C**). The scopoletin inhibitory effect was less prominent on *Pseudomonas* JAB1, whose growth was hindered only at the highest assayed concentrations (**Figure 3D**; **Supplementary Figure S6B**): 1 and 2 mM scopoletin supplies caused a decrease in biomass formation (**Figure 3E**) and a reduction in the bacterial maximum growth rate (**Figure 3F**). On the other hand, *P. xenovorans* LB400 growth was unaffected by scopoletin (**Supplementary Figure S6C**).

To counteract the deleterious effects of toxic metabolites, a bacterial protection strategy is embedding cells in a biofilm matrix. Therefore, we further verified if scopoletin could affect the biofilm formation ability in PCB-degrading model bacteria. In agreement with the observed reduction in bacterial growth, biofilm formation was stimulated in *Acinetobacter* P320 when exposed at the highest assayed concentration (**Figure 3G**) and in *Pseudomonas* JAB1 at 5 and 500 µM (**Figure 3H**), while a reduction in the ability to build the biofilm matrix was observed in *Paraburkholderia* LB400 (**Figure 3I**).

As with scopoletin, β-alanine also showed a significant reduction in the root exudation profile of PCB-18-stressed plantlets. It was observed that at the highest assayed concentration, β-alanine stimulated *Paraburkholderia* LB400 biofilm formation, potentially pointing to a deleterious effect for bacterial cells (**Supplementary Figure S7**).

### 3.4 Scopoletin negatively affected the plant-growth-promoting ability of *Acinetobacter* P320, but not the degradative potential of *Pseudomonas* JAB1 and *Paraburkholderia* LB400

As a result of the “cry-for-help” hypothesis, it is expected that the *Arabidopsis* interaction with PCB-degrading bacteria would lead to improved plant resistance to the deleterious effect induced by these pollutants. It was previously observed that strain LB400 could improve plant growth both under control conditions and when plants were challenged with 20 µM PCB-18 (Ghitti et al., 2024). A similar trend was also observed for *Pseudomonas* JAB1 (**Supplementary Figure S8**).

**Figure 4:**
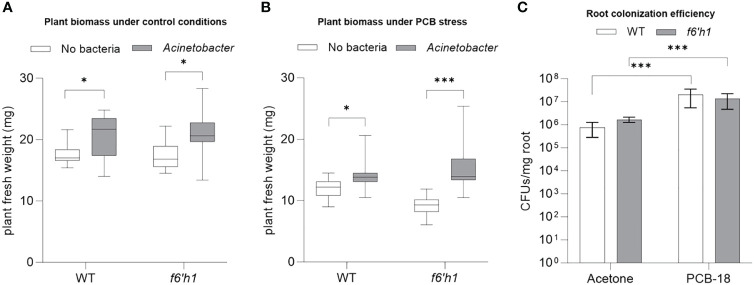
Scopoletin reduced *Acinetobacter* P320-triggered plant growth promotion under polychlorinated biphenyl (PCB) stress. **(A)** The fresh weight of *Arabidopsis* plantlets exposed to mock treatment with acetone showed that *Acinetobacter* P320 is able to trigger a similar degree of growth promotion in both WT and the scopoletin-depleted *f6’h1* line. *:*p* ≤ 0.05. **(B)** The fresh weight of *Arabidopsis* plantlets stressed by treatment with 20 µM PCB-18 indicated that *Acinetobacter* P320-induced growth promotion is enhanced in *f6’h1* plantlets (83%) compared to WT (18%), suggesting that scopoletin interferes with the bacterial plant growth promotion under PCB stress. Statistical analysis was performed by applying the Mann–Whitney test (*n* = 21). *:*p* ≤ 0.05; ***:*p* ≤ 0.001. **(C)** Root colonization efficiency of *Acinetobacter* P320 in WT and *f6’h1* plantlets exposed to mock treatment or to PCB-18 stress. Statistical analysis was performed by applying the Mann–Whitney test (three biological replicates with three technical replicates each). ***:*p* ≤ 0.001.

Considering also our previous observations that indicated *Acinetobacter* P320 as the most sensitive strain to scopoletin, we investigated scopoletin influence on *Acinetobacter* P320–plant interaction under PCB-18 stress by comparing the outcome of the bacterium association with *Arabidopsis* WT *versus* the *f6’h1* mutant that is impaired in scopoletin biosynthesis. In mock-treated plantlets, *Acinetobacter* P320 stimulated plant growth in both WT and *f6’h1* lines without significant differences (**Figure 4A**; **Supplementary Figure S9**), suggesting that the bacterial strain has a plant growth promotion potential *per se*. Upon PCB-18 stress, *Acinetobacter* P320 was able to boost plant growth, enhancing plant resistance to PCB stress (**Figure 4B**). Importantly, in the presence of PCB-18, the *Acinetobacter* P320-triggered growth promotion was enhanced in scopoletin-depleted plants (+83%) compared to the wild-type ones (+18%) (**Figure 4B**; **Supplementary Figure S9**), although colonization efficiency was not altered in the two genotypes (**Figure 4C**). These results indicate that scopoletin negatively affected *Acinetobacter* P320-promoted beneficial effect on the plant under PCB stress.

On the other hand, scopoletin did not affect the degradative functionality of *Pseudomonas* JAB1 and *Paraburkholderia* LB400, being able to trigger the expression of the *bph* operon in the two strains similarly to the positive inducer biphenyl (**Supplementary Figures S10A, B**).

### 3.5 Metabolites whose accumulation was enhanced under PCB stress serve as nutrients for PCB-degrading bacteria and diversely affected rhizocompetence traits

**Figure 5:**
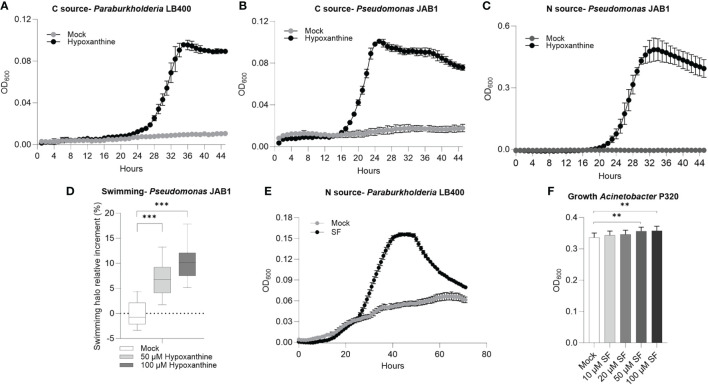
Hypoxanthine and L-seryl-L-phenylalanine (SF) are exploited as nutrient sources and signaling molecules by polychlorinated biphenyl-degrading bacteria. **(A, B)** Growth curves of strain LB400 and strain JAB1, respectively, in mineral medium in the presence of hypoxanthine as the carbon source. **(C)** Growth curve of strain JAB1 in mineral medium in the presence of hypoxanthine as the nitrogen source. **(D)** Swimming ability of strain JAB1 in the presence of hypoxanthine. Statistical analysis was carried out on at least three independent experiments using Dunn’s *post-hoc* test. ***:*p* ≤ 0.001. **(E)** Growth curve of strain LB400 in mineral medium in the presence of L-seryl-L-phenylalanine as the nitrogen source. **(F)** Growth modulation activity of L-seryl-L-phenylalanine supplemented as pure chemicals to 1/10 TSB liquid medium in *Acinetobacter* P320. In the graph, bacterial biomass reached at stationary phase at 23 h is reported, expressed as OD_600_. The bars represent the average ± standard deviation of three independent replicates. Statistical analysis was performed using the Mann–Whitney test by comparing the exudate concentrations with the respective solvent control. **:*p* ≤ 0.01.

Based on their increase in relative abundance in PCB-treated REs, it was verified that hypoxanthine, L-seryl-L-phenylalanine, and L-arginyl-L-valine could act as carbon/nitrogen sources, proliferation-stimulating molecules, or signaling compounds that affect the physiology of the selected degrading bacteria. Hypoxanthine was exploited as carbon source by *Paraburkholderia* LB400 (**Figure 5A**) and by *Pseudomonas* JAB1 (**Figure 5B**). In the latter strain, this compound also provided a nitrogen source for growth (**Figure 5C**) and enhanced bacterial swimming motility (**Figure 5D**). The dipeptide L-seryl-L-phenylalanine (SF) was used by strain LB400 as nitrogen source (**Figure 5E**) and acted as a growth-promoting factor by enhancing *Acinetobacter* P320 biomass formation and total proliferation when added as a supplement in a rich growth medium at the highest concentrations of 50 and 100 µM (**Figure 5F**). Biofilm formation showed contrasting results depending on the bacterial strain, the root exudate compounds, and assayed concentrations (**Supplementary Figure S11**).

## 4 Discussion

Rhizoremediation exploits the plant holobiont services to catabolize the pollutants and is a sustainable solution particularly suited for large areas contaminated by PCBs. This technology is based on the beneficial interaction established by plants and their associated microbiomes. In particular, root exudation performs a biostimulating role by providing nutrients, signaling molecules, and co-metabolites to significantly increase the biological activity of degrading microorganisms (Lu et al., 2017; Vaidya et al., 2022). On the other side, through their catabolism, degrading microorganisms contribute to decrease in the rhizosphere the concentrations of these detrimental xenobiotics, which the plant cannot remove efficiently due to the lack of specific detoxification systems (Huang et al., 2022). In fact, although the so-called green liver model is designated to detoxify root-absorbed PCBs through oxidation, conjugation with glutathione, and storage in the vacuole (Coleman et al., 1997), the system is inefficient (Schäffner et al., 2002) because PCB catabolism *in planta* is hampered by several factors that can dramatically influence their recalcitrance (Rezek et al., 2007; Van Aken et al., 2010).

In this study, we explored the hypothesis that the PCB-induced phytotoxic effects could modify the root chemistry in a “cry-for-help” strategy to feed and sustain PCB-degrading microbes in the rhizosphere. By using *Arabidopsis thaliana*, a model plant for holobiont studies (Poupin et al., 2023), we developed an experimental setup to reproduce the main hallmarks of PCB phytotoxicity, including reduced plant growth (Subramanian et al., 2018), chlorosis (Liu and Schnoor, 2008), overproduction of ROS (Wang et al., 2021), and alteration of plant homeostasis (Ren et al., 2022). REs collected at T2 were selected as representatives of the *bona fide* PCB-triggered shift in root chemistry based on (i) enhanced proliferation of strain LB400 by exploiting the root exudates released by PCB-exposed plantlets compared to the ones exuded by plants in the mock treatment and (ii) the increased expression levels of PCB-stress- and flavonoid-responsive genes. The metabolomic investigation performed in this work demonstrated that the root exudation pattern was dramatically altered under PCB-18 stress compared to mock-treated plantlets, potentially supporting the “cry-for-help” hypothesis triggered by PCB’s detrimental effect. Plant metabolic and physiological responses to xenobiotic exposure, including PCBs, are still poorly characterized (Subramanian et al., 2018; Lin et al., 2020) and would deserve increased attention from the research community to drive knowledge-based solutions for rhizoremediation in terms of plant species selection in order to cope with stress and biostimulate PCB degradation as observed for *Festuca arundinacea* in a semi-field trial (Terzaghi et al., 2019).

Most of the metabolites detected in the analysis could not be identified despite the technical improvement of metabolomics approaches in recent years (Patel et al., 2021). The expected repertoire of metabolites in plants is extremely complex and uncharted (Alseekh and Fernie, 2018), pushing the limits in terms of molecule identification (Aharoni et al., 2023). The annotated metabolites detected in *Arabidopsis* REs included not only organic acids (like pimelic, citric, malic, and azelaic acids), many amino acids and short peptides, sugars (including galactose and raffinose), purines, and pyrimidines (like adenine, uracil) but also osmolytes (like betaine and choline). Despite being acknowledged as key co-metabolites in PCB degradation, the flavonoid compounds kaempferol, dihydrokaempferol, and genistein 7-O-beta-D-glucoside, which were annotated in the metabolome analysis, did not show a significant variation in their relative abundance among treatments. No other flavonoids were detected in the experimental setup of the present study: REs collection at longer time points or plantlets exposition to different PCB congeners or to a higher dosage would potentially lead to uncover new PCB-triggered exudates and possibly also other specialized metabolites. Glucosinolates and their breakdown products, for instance, are characteristic metabolites of the Brassicaceae and were shown to strongly affect the taxonomical composition of *Arabidopsis* rhizosphere microbiome, with specific taxa enrichment profiles, including *Flavobacterium* and an unclassified *Diplorickettsiaceae* (Chroston et al., 2024). Leaf-spraying of silver nitrate acted as a powerful elicitor for *Arabidopsis* secondary metabolites including indole glucosinolates, scopoletin, camalexin, and flavonoids like quercetin and kaempferol, highlighting a crosstalk among defense metabolites, although the mechanisms are still elusive (Cañizares et al., 2024). Therefore, the investigation of potential additive, synergistic, or antagonistic effects among *Arabidopsis*-specialized metabolites deserves further attention to verify their modulatory role on PCB-degrading microorganisms.

To uncover the possible impact of the identified metabolites on the physiology and functionality of PCB-degrading microorganisms, three bacteria previously isolated from contaminated sites were selected, which belong to different bacterial genera that are associated to the *Arabidopsis* holobiont (Poupin et al., 2023). *Burkholderiaceae* has been described as an important component of the *Arabidopsis* floral microbiome and comprises beneficial bacteria able to support plant growth and resistance to abiotic stresses (Massoni et al., 2021; Pal et al., 2022). The *Acinetobacter* genus was described for its ability to contribute to the plant phosphorus budget by phosphate solubilization activities (Yaghoubi Khanghahi et al., 2021), while the genus *Pseudomonas* accounts for highly versatile bacteria showing biocontrol, plant-growth-promoting potential and pollutant degradative abilities (Kuhl-Nagel et al., 2022). In our study, it was shown that the three assayed strains were able to establish a beneficial association with *Arabidopsis* by colonizing the root system, stimulating plant growth under control conditions, and boosting plant resistance to PCB stress.

Among the identified compounds, scopoletin is one of the best characterized *Arabidopsis* exudate components (Stringlis et al., 2019): it is produced under iron deficiency and it selectively impacts the assembly of the rhizosphere microbial community (Voges et al., 2019), leading to enhanced plant growth due to the generation of conditions that favor the establishment of iron-mobilizing bacteria like *Pseudomonas simiae* WCS417 (Stringlis et al., 2018). The ability of scopoletin to sculpt the root-associated microbiome is linked to its differential antimicrobial features, inhibiting the growth of phytopathogenic fungi like *Fusarium* and *Verticilloides* spp. while being ineffective on iron-mobilizing bacteria like *Pseudomonas capeferrum* WCS358 (Stringlis et al., 2018), supposedly insensitive to its ROS-induced stress (Voges et al., 2019). In our study, we observed that, in PCB-18-stressed plants, scopoletin relative abundance decreased in the REs, and this led to the hypothesis that this reduction might be linked to the antimicrobial properties of this compound, affecting the proliferation and vitality of PCB-degrading bacteria. Indeed it was observed that both *Acinetobacter* P320 and *Pseudomonas* JAB1 were sensitive to scopoletin antimicrobial activity, although at different levels, while only *P. xenovorans* LB400 was resistant. Scopoletin negatively affected the different growth parameters of sensitive bacterial strains, decreasing their viability, biomass accumulation, and maximum growth rate with the ultimate effect to unbalance their fitness and the services provided to the plant. Notably, for *Acinetobacter* P320, it was observed that the bacterium-triggered enhancement of plant growth under PCB-18 stress was restricted by scopoletin, as indicated by the higher plant growth promotion effect in the scopoletin-depleted mutant *f6’h1* compared to scopoletin-producing WT plantlets. However, the functionality of *Pseudomonas* JAB1 in terms of PCB catabolism was not compromised by scopoletin: similarly to umbelliferone, another coumarin compound (Zubrova et al., 2021), scopoletin could induce the expression of the *bph*-encoded degradative pathway. In *Paraburkholderia* LB400, flavone and quercetin were demonstrated to act as *bph* inducer (Ghitti et al., 2024), while this is the first report for a coumarin to perform such activity. The comprehension of the factors affecting the degradative functionality in PCB degradative bacteria is still poorly characterized, also considering the lack of experimental data collected by using REs with a complex blend. Bacterial cell adhesion in corn kernel biochar, for instance, seems to promote strain LB400 viability and induce a higher expression of the *bph-*encoded dioxygenases (Dong et al., 2024).

Furthermore, coumarins have been identified as effective antibiofilm and anti-quorum sensing compounds in medical/pharmaceutical fields against human pathogens (Reen et al., 2018), while few reports are available in the environmental microbiology context as antagonist molecules able to block *Ralstonia solanacearum* outbreak (Yang et al., 2016, 2017). Being sensitive to scopoletin, in the presence of this compound, *Acinetobacter* P320 and *Pseudomonas* JAB1 showed an increased ability to form biofilm, which could potentially act as a barrier against penetration and contact with toxic compounds (Shree et al., 2023).

To date, scopoletin’s role in plant–microbe interactions has been mainly documented for iron homeostasis, with important implications for phytopathogen control (Stringlis et al., 2019), while, to our knowledge, this is the first report about scopoletin modulation in plants exposed to xenobiotic stress. Collectively, these findings indicate a regulated release of scopoletin by *Arabidopsis* roots exposed to different stresses, like iron deficiency and PCB pollution, reinforcing its mechanisms of action in modulating the composition of the root-associated microbial community through its antimicrobial ability. The decreased abundance of scopoletin under PCB-18 stress, due to its inhibitory effect on the performance of some PCB-degrading bacteria, may be instrumental to generate more suitable conditions in the rhizosphere niche to accommodate scopoletin-sensitive degrading bacteria.

β-alanine is part of the 250 non-proteinogenic amino acids produced by plants to perform anti-herbivory and anti-microbial actions and to mediate response to abiotic stresses (Parthasarathy et al., 2019). In the LC–MS analysis, alpha and beta isomers cannot be separated; therefore, the presence of the alpha form cannot be excluded. The accumulation of both forms is documented under a diverse array of stresses, including drought and heavy metal contamination for β-alanine (Parthasarathy et al., 2019) and waterlogging in wheat plants for α-alanine (Cid et al., 2024). The decrease of N-hydroxyethyl-β-alanine relative abundance in REs under PCB-18 stress may appear controversial. Nevertheless, β-alanine is a structural component of pantothenate that contributes to coenzyme A backbone. The TCA cycle, in which the coenzyme A shuttle is fundamental, is described as a major target of PCB stress in rice. Exposure to methoxylated PCB derivatives mainly affected the rice saccharide catabolism, including pyruvate metabolism and the TCA cycle, resulting in a greater energy consumption that dramatically reduced plant growth (Lin et al., 2020). Therefore, the decrease of the β-alanine compound in PCB-exposed plantlets may be part of the plant physiology adaptation to PCB stress. Indeed the N-hydroxyethyl-β-alanine-mediated effect on the physiology of PCB-degrading bacteria was largely cryptic, and the only effect that we observed was related to *P. xenovorans* LB400 decreased biofilm formation at the intermediate assayed concentration. Since biofilm formation on the rhizoplane is considered an early determinant in successful plant–microbe interactions, the fitness of strain LB400 could be negatively affected by N-hydroxyethyl-β-alanine.

The identified compounds that increased their relative abundance in REs upon PCB-18 treatment were all primary metabolites, an abundant and heterogenous class of molecules that are demonstrated to exert a strong selective pressure on plant and soil microbial diversity (Shi et al., 2011; Steinauer et al., 2016) through their preferential microbial utilization (Zhalnina et al., 2018).

Hypoxanthine is a purine catabolic product that is then converted to allantoin and allantoate, metabolites that can act as ROS scavengers and were shown to attenuate stress symptoms under darkness stress and senescence in *Arabidopsis* (Brychkova et al., 2008). Its overproduction in REs in the presence of PCB-18 can be interpreted as part of the “cry-for-help” plant response to recruit PCB-degrading bacteria. In the rhizosphere, hypoxanthine is generally used by soil microorganisms as a nitrogen source (Izaguirre-Mayoral et al., 2018), and a specific chemoreceptor for its translocation has been identified in *P. putida* KT2440 (Fernández et al., 2016). In our study, it was observed that hypoxanthine can be exploited as both N and C sources by *Pseudomonas* JAB1 and that it could, moreover, improve bacterial swimming motility. Hypoxanthine depletion in *Pseudomonas fluorescens* Pf0–1 led to a reduced performance in the rhizosphere due to an altered transition from planktonic cells to biofilm-attached cells (Yoshioka and Newell, 2016). In our study, hypoxanthine inhibition of biofilm formation for *Pseudomonas* JAB1 and *Paraburkholderia* LB400 was concentration-dependent. Overall, these results indicate that hypoxanthine can be exploited as a nutrient and can diversely affect rhizosphere colonization traits in the selected PCB-degrading bacteria.

Low-molecular-weight peptides, amino acids, and derivatives produced from the cleavage of larger proteins are typical constituents of plant root exudation (McLaughlin et al., 2023), and different dipeptides were identified in *Arabidopsis* root secretome (Strehmel et al., 2014). In our work, the identified dipeptides overproduced under PCB stress showed a capacity both to serve as nitrogen sources and as stimulators of bacterial proliferation as putative co-metabolites, in agreement with previous findings (Moormann et al., 2022). Furthermore, we suggested a possible role as mediators of root colonization processes like biofilm formation and swimming motility, aspects in which peptide regulatory actions are largely overlooked (Minen et al., 2023). To conclude, our findings indicate that the metabolites that are more abundant in *Arabidopsis* REs under PCB-18 stress may contribute to sustain the growth, proliferation, and rhizosphere colonization of PCB-degrading bacteria, with effects that are species-specific and concentration dependent.

The investigation on the role played by the identified REs on degradative bacteria has been performed by adopting *in vitro* tests and, depending on the assay, a well-established metabolite concentration. Unfortunately, quantitative analyses on the amount of REs in the rhizosphere are sparse, although it is known that metabolite abundance declines with increased distance from root surfaces (Eze and Amuji, 2024). Moreover, the impact of abiotic processes like adsorption to soil particles, biological degradation by the soil microbiota, and the half-life of the different compounds should be taken into consideration (Shaw et al., 2006; Del Valle et al., 2020). Therefore, it is not possible to exclude that the concentration used for the *in vitro* experiments are higher than those in the rhizosphere. Anyway, in a hydroponic root exudate collection system, flavonoids were estimated in micromolar ranges (Toussaint et al., 2012), suggesting that the concentrations used in the present study are well suited to investigate their effect on bacterial physiology.

To strengthen the relevance of the findings of the present study, which shed light on the partnership between host plant and degrading bacteria in presence of PCBs, alternative root exudate collection methods may be crucial to simulate field-like settings—for instance, leachate collection of three grassland species cultivated in pots allowed the identification of many species-specific exuded metabolites (Williams et al., 2021). On the other hand, complications often arise in data interpretation of root exudate collection from soil-cultivated plants compared to those collected in hydroponic and liquid media. Among them, there is the risk of sample contamination from soil chemical inputs, the presence of partially catabolized exudate by microbes and of metabolites produced by microorganisms (Pantigoso et al., 2020).

There are still several open questions about the ecology of rhizoremediation that would benefit from the strengthening of the holobiont concept in the approaches adopted for xenobiotic cleanup. A crucial point is represented, for instance, by the choice of the plant species: among those commonly used for bioremediation purposes, they are often selected for their ability to tolerate PCBs at high concentrations or to bio-accumulate them in root and stem tissues, like pumpkins and zucchini (Aslund et al., 2007, 2008). The contribution of the associated microbiomes to these phenotypic traits is largely overlooked, although the plant association with degrading microorganisms is fundamental for effective remediation (Wang et al., 2023). By modulating root exudation, plants can generate a stimulating niche to recruit beneficial bacteria, directly contributing to enhanced plant fitness (Rohrbacher and St-Arnaud, 2016), or can improve PCB bio-availability to be further catabolized by degrading microorganisms (Terzaghi et al., 2021). Novel knowledge on these issues will provide a broader and more efficient application of rhizoremediation on PCB-contaminated environments.

## 5 Conclusion

The analyses, performed on the developed *in vitro* setup that reproduce PCB phytotoxic effects, delineate the scenario of a shift in *Arabidopsis* exudation pattern induced by xenobiotic stress. Our efforts to characterize the role of PCB-18-modulated metabolites on the physiology of three PCB-degrading bacteria could not rule out a univocal framework since some effects were either species-specific or showed differences based on the analyzed functional traits. Indeed, although rhizodeposition is acknowledged as a crucial player in microbiome dynamics, contrasting effects exerted by pure root metabolites, depending on their concentration and on the bacterial species involved, can hamper the comprehension of clear causation (Huang et al., 2019; He et al., 2022; Ghitti et al., 2024). The use of synthetic communities rather than single bacterial isolates could contribute to a more comprehensive understanding of the influence played by root metabolites on the diversity and activity of the plant-associated microbiome (Li et al., 2024). Nevertheless, the collected data suggest a potential plant “cry-for-help” event in response to PCB-18 exposure in terms of root chemistry remodeling. The role played by the differentially exuded compounds could be investigated only by using the five annotated metabolites, and based on these findings, they seem to be potentially involved in the accommodation of degrading bacteria in the rhizosphere by decreasing those exudates that can hamper their growth and performance (scopoletin and N-hydroxyethyl-β-alanine) and increasing the relative abundance of compounds that can be exploited as nutrients and signaling molecules for rhizocompetence. Based on the evidence obtained in the standardized setup of this investigation, further studies are claimed since the identification of the root metabolic drivers of bacterial assembly in polluted environments is of paramount importance to steer the soil microbiome, potentially enriching those populations endowed with the highest bioremediation potential.

## Data availability statement

The datasets presented in this study can be found in online repositories. The names of the repository/repositories and accession number(s) can be found below: UNIMI Dataverse repository at https://dataverse.unimi.it/dataverse/SENSE.

## Author contributions

ER: Conceptualization, Data curation, Formal analysis, Funding acquisition, Investigation, Methodology, Project administration, Supervision, Validation, Visualization, Writing – original draft, Writing – review & editing. EG: Data curation, Formal analysis, Investigation, Methodology, Validation, Visualization, Writing – review & editing. FM: Formal analysis, Visualization, Writing – review & editing. SB: Conceptualization, Funding acquisition, Project administration, Resources, Supervision, Writing – review & editing.

## References

[B1] Afridi M. S. Kumar A. Javed M. A. Dubey A. de Medeiros F. H. V. Santoyo G. (2024). Harnessing root exudates for plant microbiome engineering and stress resistance in plants. Microbiol. Res. 279, 127564. doi: 10.1016/j.micres.2023.127564 38071833

[B2] Aharoni A. Goodacre R. Fernie A. R. (2023). Plant and microbial sciences as key drivers in the development of metabolomics research. PNAS 120, 1–11. doi: 10.1073/pnas.2217383120 PMC10041103 36930598

[B3] Alseekh S. Fernie A. R. (2018). Metabolomics 20 years on: what have we learned and what hurdles remain? Plant J. 94, 933–942. doi: 10.1111/tpj.13950 29734513

[B4] Anderson M. J. Gorley R. N. Clarke K. R. (2008). PERMANOVA+ for primer: Guide to software and statistical methods (Plymouth, UK: PRIMER-E).

[B5] Asai K. Takagib K. Shimokawab M. Sueb T. Hibib A. Hirutab T.. (2002). Phytoaccumulation of coplanar PCBs by *Arabidopsis thaliana* . Environ. pollut. 120, 509–511. doi: 10.1016/S0269-7491(02)00311-1 12442774

[B6] Aslund M. L. W. Rutter A. Reimer K. J. Zeeb B. A. (2008). The effects of repeated planting, planting density, and specific transfer pathways on PCB uptake by *Cucurbita pepo* grown in field conditions. Sci. Total Environ. 405, 14–25. doi: 10.1016/j.scitotenv.2008.07.066 18786697

[B7] Aslund M. L. W. Zeeb B. A. Rutter A. Reimer J. K. (2007). *In situ* phytoextraction of polychlorinated biphenyl—(PCB) contaminated soil. Sci. Total Environ. 374, 1–12. doi: 10.1016/j.scitotenv.2006.11.052 17258285

[B8] Bao L. Gao C. Li M. Chen Y. Lin W. Yang Y.. (2013). Biomonitoring of non-dioxin-like polychlorinated biphenyls in transgenic *Arabidopsis* using the mammalian pregnane X receptor system: A role of pectin in pollutant uptake. PloS One 8, e79428. doi: 10.1371/journal.pone.0079428 24236133 PMC3827382

[B9] Bartolini M. Grau R. (2019). Assessing different ways of *Bacillus subtilis* spreading over abiotic surfaces. Bio Protoc. 9, e3425. doi: 10.21769/BioProtoc.3425 PMC7853940 33654922

[B10] Berendsen R. L. Vismans G. Yu K. Song Y. de Jonge R. Burgman W. P.. (2018). Disease-induced assemblage of a plant-beneficial bacterial consortium. ISME J. 12, 1496–1507. doi: 10.1038/s41396-018-0093-1 29520025 PMC5956071

[B11] Brychkova G. Fluhr R. Sagi M. (2008). Formation of xanthine and the use of purine metabolites as a nitrogen source in *Arabidopsis* plants. Plant Signal Behav. 3, 999–1001. doi: 10.4161/psb.6304 19704433 PMC2633756

[B12] Cañizares E. Acién J. M. Gumuş B. Ö. Vives-Peris V. González-Guzmán M. Arbona V. (2024). Interplay between secondary metabolites and plant hormones in silver nitrate-elicited *Arabidopsis thaliana* plants. Plant Physiol. Biochem. 208, 108483. doi: 10.1016/j.plaphy.2024.108483 38457948

[B13] Chain P. S. G. Denef V. J. Konstantinidis K. T. Vergez L. M. Agulló L. Reyes V. L.. (2006). *Burkholderia xenovorans* LB400 harbors a multi-replicon, 9.73-Mbp genome shaped for versatility. PNAS 103, 15280–15287. doi: 10.1073/pnas.0606924103 17030797 PMC1622818

[B14] Chroston E. C. M. Bziuk N. Stauber E. J. Ravindran B. M. Hielscher A. Smalla K.. (2024). Plant glucosinolate biosynthesis and breakdown pathways shape the rhizosphere bacterial/archaeal community. Plant Cell Environ. 47 (6), 2127–2145. doi: 10.1111/pce.14870 38419355

[B15] Cid A. G. Francioli D. Kolb S. Tandron Moya Y. A. von Wirén N. Hajirezaei M.-R. (2024). Transcriptomic and metabolomic approaches elucidate the systemic response of wheat plants under waterlogging. J. Exp. Bot. 75, 1510–1529. doi: 10.1093/jxb/erad453 38014629

[B16] Coleman J. Blake-Kalff M. Davies E. (1997). Detoxification of xenobiotics by plants: chemical modification and vacuolar compartmentation. Trends Plant Sci. 2, 144–151. doi: 10.1016/S1360-1385(97)01019-4

[B17] Daudi A. O’Brien J. A. (2012). Detection of hydrogen peroxide by DAB staining in *Arabidopsis* leaves. Bio Protoc. 2, 4–7. doi: 10.21769/BioProtoc.263 PMC4932902 27390754

[B18] Del Valle I. Webster T. M. Cheng H.-Y. Thies J. E. Kessler A. Miller M. K.. (2020). Soil organic matter attenuates the efficacy of flavonoid-based plant-microbe communication. Sci. Adv. 6, eaax8254. doi: 10.1126/sciadv.aax8254 32064339 PMC6989149

[B19] Ding Y. Ren H. Hao X. Zhang R. Hao J. Liu J.. (2024). Enhanced phytoremediation of PCBs-contaminated soil by co-expressing *tfdB* and *bphC* in *Arabidopsis* aiding in metabolism and improving toxicity tolerance. Environ. Exp. Bot. 217, 105548. doi: 10.1016/j.envexpbot.2023.105548

[B20] Dong Q. LeFevre G. H. Mattes T. E. (2024). Black carbon impacts on *Paraburkholderia xenovorans* strain LB400 cell enrichment and activity: implications toward lower-chlorinated polychlorinated biphenyls biodegradation potential. Environ. Sci. Technol. 58, 3895–3907. doi: 10.1021/acs.est.3c09183 38356175 PMC10902836

[B21] Eze M. E. Amuji C. G. (2024). Elucidating the significant roles of root exudates in organic pollutant biotransformation within the rhizosphere. Sci. Rep. 14, 2359. doi: 10.1038/s41598-024-53027-x 38286879 PMC10824751

[B22] Fan T. Yang L. Wu X. Ni J. Jiang H. Zhang Q.. (2016). The PSE1 gene modulates lead tolerance in *Arabidopsis* . J. Exp. Bot. 67, 4685–4695. doi: 10.1093/jxb/erw251 27335453 PMC4973742

[B23] Feng H. Fu R. Luo J. Hou X. Gao K. Su L.. (2023). Listening to plant’s Esperanto via root exudates: reprogramming the functional expression of plant growth-promoting rhizobacteria. New Phytol. 239, 2307–2319. doi: 10.1111/nph.19086 37357338

[B24] Fernández M. Morel B. Corral-Lugo A. Krell T. (2016). Identification of a chemoreceptor that specifically mediates chemotaxis toward metabolizable purine derivatives. Mol. Microbiol. 99, 34–42. doi: 10.1111/mmi.13215 26355499

[B25] Ghitti E. Rolli E. Crotti E. Borin S. (2022). Flavonoids are intra- and inter-kingdom modulator signals. Microorganisms 10, 2479. doi: 10.3390/microorganisms10122479 36557733 PMC9781135

[B26] Ghitti E. Rolli E. Vergani L. Borin S. (2024). Flavonoids influence key rhizocompetence traits for early root colonization and PCB degradation potential of *Paraburkholderia xenovorans* LB400. Front. Plant Sci. 15. doi: 10.3389/fpls.2024.1325048 PMC10869545 38371405

[B27] Hao D. Wang H. Niu L. (2020). Activation of six lipocalins genes’ transcription under PCB18 stress in OsTIL-silenced *Oryza sativa* L. Ecotoxicol. Environ. Saf. 204, 111063. doi: 10.1016/j.ecoenv.2020.111063 32791358

[B28] Harbort C. J. Hashimoto M. Inoue H. Niu Y. Guan R. Rombolà A. D.. (2020). Root-secreted coumarins and the microbiota interact to improve iron nutrition in *Arabidopsis* . Cell Host Microbe 28, 825–837.e6. doi: 10.1016/j.chom.2020.09.006 33027611 PMC7738756

[B29] He D. Singh S. K. Peng L. Kaushal R. Vílchez J. I. Shao C.. (2022). Flavonoid-attracted Aeromonas sp. from the *Arabidopsis* root microbiome enhances plant dehydration resistance. ISME J. 16, 2622–2632. doi: 10.1038/s41396-022-01288-7 35842464 PMC9561528

[B30] Huang A. C. Jiang T. Liu Y.-X. Bai Y.-C. Reed J. Qu B.. (2019). A specialized metabolic network selectively modulates *Arabidopsis* root microbiota. Science 364, eaau6389. doi: 10.1126/science.aau6389 31073042

[B31] Huang Z. Jiang L. Lu W. Luo C. Song M. (2022). *Elsholtzia splendens* promotes phenanthrene and polychlorinated biphenyl degradation under Cu stress through enrichment of microbial degraders. J. Hazard. Mater. 438, 129492. doi: 10.1016/j.jhazmat.2022.129492 35803192

[B32] Izaguirre-Mayoral M. L. Lazarovits G. Baral B. (2018). Ureide metabolism in plant-associated bacteria: purine plant-bacteria interactive scenarios under nitrogen deficiency. Plant Soil 428, 1–34. doi: 10.1007/s11104-018-3674-x

[B33] Jin X. Shuai J. Peng R. Zhu B. Fu X. Tian Y.. (2011). Identification of candidate genes involved in responses of *Arabidopsis* to polychlorinated biphenyls based on microarray analysis. Plant Growth Regul. 65, 127–135. doi: 10.1007/s10725-011-9582-1

[B34] Kearns D. B. (2010). A field guide to bacterial swarming motility. Nat. Rev. Microbiol. 8, 634–644. doi: 10.1038/nrmicro2405 20694026 PMC3135019

[B35] Kuhl-Nagel T. Rodriguez P. A. Gantner I. Chowdhury S. P. Schwehn P. Rosenkranz M.. (2022). Novel pseudomonas sp. SCA7 promotes plant growth in two plant families and induces systemic resistance in *Arabidopsis thaliana* . Front. Microbiol. 13, 923515. doi: 10.3389/fmicb.2022.923515 35875540 PMC9297469

[B36] Langlois-Meurinne M. Gachon C. M. Saindrenan P. (2005). Pathogen-responsive expression of glycosyltransferase genes UGT73B3 and UGT73B5 is necessary for resistance to *Pseudomonas syringae* pv *tomato* in *Arabidopsis* . Plant Physiol. 139, 1890–1901. doi: 10.1104/pp.105.067223 16306146 PMC1310567

[B37] Li L. Xu X. Chen C. Shen Z. (2016). Genome-wide characterization and expression analysis of the germin-like protein family in rice and *Arabidopsis* . Int. J. Mol. Sci. 17, 1622. doi: 10.3390/ijms17101622 27669230 PMC5085655

[B38] Li X. Zheng X. Yadav N. Saha S. Salama E. S. Li X.. (2024). Rational management of the plant microbiome for the Second Green Revolution. Plant Commun. 5, 100812. doi: 10.1016/j.xplc.2024.100812 38213028 PMC11009158

[B39] Lin F. Sun J. Liu N. Zhu L. (2020). Phytotoxicity and metabolic responses induced by tetrachlorobiphenyl and its hydroxylated and methoxylated derivatives in rice (*Oryza sative* L.). Environ. Int. 139, 105695. doi: 10.1016/j.envint.2020.105695 32272295

[B40] Liu J. Schnoor J. L. (2008). Uptake and translocation of lesser-chlorinated polychlorinated biphenyls (PCBs) in whole hybrid poplar plants after hydroponic exposure. Chemosphere 73, 1608–1616. doi: 10.1016/j.chemosphere.2008.08.009 18793792 PMC2668963

[B41] Lu H. Sun J. Zhu L. (2017). The role of artificial root exudate components in facilitating the degradation of pyrene in soil. Sci. Rep. 7, 7130. doi: 10.1038/s41598-017-07413-3 28769098 PMC5541004

[B42] McLaughlin S. Zhalnina K. Kosina S. Northen T. R. Sasse J. (2023). The core metabolome and root exudation dynamics of three phylogenetically distinct plant species. Nat. Commun. 14, 1649. doi: 10.1038/s41467-023-37164-x 36964135 PMC10039077

[B43] Massoni J. Bortfeld-Miller M. Widmer A. Vorholt J. A. (2021). Capacity of soil bacteria to reach the phyllosphere and convergence of floral communities despite soil microbiota variation. Proc. Natl. Acad. Sci. U.S.A. 118 (41), e2100150118. doi: 10.1073/pnas.2100150118 34620708 PMC8521660

[B44] Minen R. I. Thirumalaikumar V. P. Skirycz A. (2023). Proteinogenic dipeptides, an emerging class of small-molecule regulators. Curr. Opin. Plant Biol. 75, 102395. doi: 10.1016/j.pbi.2023.102395 37311365

[B45] Moormann J. Heinemann B. Hildebrandt T. M. (2022). News about amino acid metabolism in plant–microbe interactions. Trends Biochem. Sci. 47, 839–850. doi: 10.1016/j.tibs.2022.07.001 35927139

[B46] Musilova L. Ridl J. Polivkova M. Macek T. Uhlik O. (2016). Effects of secondary plant metabolites on microbial populations: changes in community structure and metabolic activity in contaminated environments. Int. J. Mol. Sci. 17, 1205. doi: 10.3390/ijms17081205 27483244 PMC5000603

[B47] Narasimhan K. Basheer C. Bajic V. B. Swarup S. (2003). Enhancement of plant-microbe interactions using a rhizosphere metabolomics-driven approach and its application in the removal of polychlorinated biphenyls. Plant Physiol. 132, 146–153. doi: 10.1104/pp.102.016295 12746520 PMC166960

[B48] Navarro-Pérez M. L. Fernández-Calderón M. C. Vadillo-Rodríguez V. (2022). Decomposition of growth curves into growth rate and acceleration: a novel procedure to monitor bacterial growth and the time-dependent effect of antimicrobials. Appl. Environ. Microbiol. 88, e01849–e01821. doi: 10.1128/aem.01849-21 34878817 PMC8824196

[B49] Pal G. Saxena S. Kumar K. Verma A. Sahu P. K. Pandey A.. (2022). Endophytic *Burkholderia*: Multifunctional roles in plant growth promotion and stress tolerance. Microbiol. Res. 265, 127201. doi: 10.1016/j.micres.2022.127201 36167006

[B50] Pantigoso H. A. Yuan J. DiLegge M. J. Vivanco J. M. (2021). “Methods for root exudate collection and analysis," in The plant microbiome. Methods in molecular biology, vol. 2232. Eds. Carvalhais L. C. Dennis P. G. (New York, NY: Humana). doi: 10.1007/978-1-0716-1040-4_22 33161555

[B51] Pantigoso H. A. Yuan J. He Y. Guo Q. Vollmer C. Vivanco J. M. (2020). Role of root exudates on assimilation of phosphorus in young and old *Arabidopsis thaliana* plants. PloS One 15, e0234216. doi: 10.1371/journal.pone.0234216 32492072 PMC7269232

[B52] Parthasarathy A. Savka M. A. Hudson A. O. (2019). The synthesis and role of β-alanine in plants. Front. Plant Sci. 10. doi: 10.3389/fpls.2019.00921 PMC6657504 31379903

[B53] Patel M. Pandey S. Kumar M. Haque M. Pal S. Yadav N. (2021). Plants metabolome study: emerging tools and techniques. Plants 10, 2409. doi: 10.3390/plants10112409 34834772 PMC8621461

[B54] Pham T. T. M. Pino Rodriguez N. J. Hijri M. Sylvestre M. (2015). Optimizing polychlorinated biphenyl degradation by flavonoid-induced cells of the rhizobacterium *Rhodococcus erythropolis* U23A. PloS One 10, e0126033. doi: 10.1371/journal.pone.0126033 25970559 PMC4430277

[B55] Poupin M. J. Ledger T. Roselló-Móra R. González B. (2023). The *Arabidopsis* holobiont: a (re)source of insights to understand the amazing world of plant–microbe interactions. Environ. Microbiome 18, 9. doi: 10.1186/s40793-023-00466-0 36803555 PMC9938593

[B56] Rai A. Umashankar S. Rai M. Kiat L. B. Bing J. A. S. Swarup S. (2016). Coordinate regulation of metabolite glycosylation and stress hormone biosynthesis by TT8 in *Arabidopsis* . Plant Physiol. 171, 2499–2515. doi: 10.1104/pp.16.00421 27432888 PMC4972274

[B57] Reen F. J. Gutiérrez-Barranquero J. A. Parages M. L. O´Gara F. (2018). Coumarin: a novel player in microbial quorum sensing and biofilm formation inhibition. Appl. Microbiol. Biotechnol. 102, 2063–2073. doi: 10.1007/s00253-018-8787-x 29392389 PMC5814477

[B58] Rehman H. M. Nawaz M. A. Shah. Z. H. Ludwig-Müller J. Chung G. Ahmad M. Q.. (2018). Comparative genomic and transcriptomic analyses of Family-1 UDP glycosyltransferase in three Brassica species and *Arabidopsis* indicates stress-responsive regulation. Sci. Rep. 8, 1875. doi: 10.1038/s41598-018-19535-3 29382843 PMC5789830

[B59] Ren H. Ding Y. Hao X. Hao J. Liu J. Wang Y. (2022). Enhanced rhizoremediation of polychlorinated biphenyls by resuscitation-promoting factor stimulation linked to plant growth promotion and response of functional microbial populations. Chemosphere 309, 136519. doi: 10.1016/j.chemosphere.2022.136519 36210576

[B60] Rezek J. Macek T. Mackova M. Triska J. (2007). Plant metabolites of polychlorinated biphenyls in hairy root culture of black nightshade *Solanum nigrum* SNC-9O. Chemosphere 69, 1221–1227. doi: 10.1016/j.chemosphere.2007.05.090 17640705

[B61] Rizaludin M. S. Stopnisek N. Raaijmakers J. M. Garbeva P. (2021). The Chemistry of stress: understanding the ‘cry for help’ of plant roots. Metabolites 11, 357. doi: 10.3390/metabo11060357 34199628 PMC8228326

[B62] Rohrbacher F. St-Arnaud M. (2016). Root exudation: the ecological driver of hydrocarbon rhizoremediation. Agronomy 6, 19. doi: 10.3390/agronomy6010019

[B63] Rolfe S. A. Griffiths J. Ton J. (2019). Crying out for help with root exudates: adaptive mechanisms by which stressed plants assemble health-promoting soil microbiomes. Curr. Opin. Microbiol. 49, 73–82. doi: 10.1016/j.mib.2019.10.003 31731229

[B64] Rolli E. de Zélicourt A. Alzubaidy H. Karampelias M. Parween S. Rayapuram N.. (2022). The Lys-motif receptor LYK4 mediates Enterobacter sp. SA187 triggered salt tolerance in *Arabidopsis thaliana* . Environ. Microbiol. 24, 223–239. doi: 10.1111/1462-2920.15839 34951090 PMC9304150

[B65] Rolli E. Marasco R. Vigani G. Ettoumi B. Mapelli F. Deangelis M. L.. (2015). Improved plant resistance to drought is promoted by the root-associated microbiome as a water stress-dependent trait. Environ. Microbiol. 17 (2), 316–331. doi: 10.1111/1462-2920.12439 24571749

[B66] Rolli E. Vergani L. Ghitti E. Patania G. Mapelli F. Borin S. (2021). ‘Cry-for-help’ in contaminated soil: a dialogue among plants and soil microbiome to survive in hostile conditions. Environ. Microbiol. 23, 5690–5703. doi: 10.1111/1462-2920.15647 34139059 PMC8596516

[B67] Salem M. A. Jüppner J. Bajdzienko K. Giavalisco P. (2016). Protocol: a fast, comprehensive and reproducible one-step extraction method for the rapid preparation of polar and semi-polar metabolites, lipids, proteins, starch and cell wall polymers from a single sample. Plant Methods 12, 45. doi: 10.1186/s13007-016-0146-2 27833650 PMC5103428

[B68] Schäffner A. Messner B. Langebartels C. Sandermann H. (2002). Genes and enzymes for in-planta phytoremediation of air, water and soil. Acta Biotechnol. 22, 141–151. doi: 10.1002/1521-3846(200205)22:1/2<141::AID-ABIO141>3.0.CO;2-7

[B69] Serra A. Nuttens A. Larvor V. Renault D. Couée I. Sulmon C.. (2013). Low environmentally relevant levels of bioactive xenobiotics and associated degradation products cause cryptic perturbations of metabolism and molecular stress responses in *Arabidopsis thaliana* . J. Exp. Bot. 64, 2753–2766. doi: 10.1093/jxb/ert119 23645866

[B70] Shaw L. J. Morris P. Hooker J. E. (2006). Perception and modification of plant flavonoid signals by rhizosphere microorganisms. Environ. Microbiol. 8, 1867–1880. doi: 10.1111/j.1462-2920.2006.01141.x 17014487

[B71] Shi S. Richardson A. E. O’Callaghan M. DeAngelis K. M. Jones E. E. Stewart A.. (2011). Effects of selected root exudate components on soil bacterial communities. FEMS Microbiol. Ecol. 77, 600–610. doi: 10.1111/j.1574-6941.2011.01150.x 21658090

[B72] Shree P. Singh C. K. Sodhi K. K. Surya J. N. Singh D. K. (2023). Biofilms: Understanding the structure and contribution towards bacterial resistance in antibiotics. Med. Microecol. 16, 100084. doi: 10.1016/j.medmic.2023.100084

[B73] Steinauer K. Chatzinotas A. Eisenhauer N. (2016). Root exudate cocktails: the link between plant diversity and soil microorganisms? Ecol. Evol. 6, 7387–7396. doi: 10.1002/ece3.2454 28725406 PMC5513276

[B74] Strehmel N. Böttcher C. Schmidt S. Scheel D. (2014). Profiling of secondary metabolites in root exudates of *Arabidopsis thaliana* . Phytochemistry 108, 35–46. doi: 10.1016/j.phytochem.2014.10.003 25457500

[B75] Stringlis I. A. de Jonge R. Pieterse C. M. J. (2019). The Age of coumarins in plant–microbe interactions. Plant Cell Physiol. 60, 1405–1419. doi: 10.1093/pcp/pcz076 31076771 PMC6915228

[B76] Stringlis I. A. Yu K. Feussner K. de Jonge R. Van Bentum S. Van Verk M. C.. (2018). MYB72-dependent coumarin exudation shapes root microbiome assembly to promote plant health. PNAS 115, E5213–E5222. doi: 10.1073/pnas.1722335115 29686086 PMC5984513

[B77] Subramanian S. Schnoor J. L. Van Aken B. (2018). Effects of polychlorinated biphenyls (PCBs) and their hydroxylated metabolites (OH-PCBs) on *Arabidopsis thaliana* . Environ. Sci. Technol. 51, 7263–7270. doi: 10.1021/acs.est.7b01538 PMC5772893 28541669

[B78] Suman J. Sredlova K. Fraraccio S. Jerabkova M. Strejcek M. Kabickova H.. (2024). Transformation of hydroxylated polychlorinated biphenyls by bacterial 2-hydroxybiphenyl 3-monooxygenase. Chemosphere 349, 140909. doi: 10.1016/j.chemosphere.2023.140909 38070605

[B79] Suman J. Strejcek M. Zubrova A. Capek J. Wald J. Michalikova K.. (2021). Predominant biphenyl dioxygenase from legacy polychlorinated biphenyl (PCB)-contaminated soil is a part of unusual gene cluster and transforms flavone and flavanone. Front. Microbiol. 12. doi: 10.3389/fmicb.2021.644708 PMC8552027 34721309

[B80] Terzaghi E. Alberti E. Raspa G. Zanardini E. Morosini C. Anelli S.. (2021). A new dataset of PCB half-lives in soil: effect of plant species and organic carbon addition on biodegradation rates in a weathered contaminated soil. Sci. Total Environ. 750, 141411. doi: 10.1016/j.scitotenv.2020.141411 32841806

[B81] Terzaghi E. Vergani L. Mapelli F. Borin S. Raspa G. Zanardini E.. (2019). Rhizoremediation of weathered PCBs in a heavily contaminated agricultural soil: Results of a biostimulation trial in semi field conditions. Sci. Total Environ. 686, 484–496. doi: 10.1016/j.scitotenv.2019.05.458 31185397

[B82] Toussaint J.-P. Pham T. T. M. Barriault D. Sylvestre M. (2012). Plant exudates promote PCB degradation by a rhodococcal rhizobacteria. Appl. Microbiol. Biotechnol. 95, 1589–1603. doi: 10.1007/s00253-011-3824-z 22202970

[B83] Uhlik O. Musilova L. Ridl J. Hroudova M. Vlcek C. Koubek J.. (2013). Plant secondary metabolite-induced shifts in bacterial community structure and degradative ability in contaminated soil. Appl. Microbiol. Biotechnol. 97, 9245–9256. doi: 10.1007/s00253-012-4627-6 23250224

[B84] Vaidya B. P. Hagmann D. F. Haramuniz J. Krumins J. A. Goodey N. M. (2022). Artificial root exudates restore microbial functioning in a metal contaminated, barren, inactive soil. Environ. pollut. 312, 120007. doi: 10.1016/j.envpol.2022.120007 35998773

[B85] Van Aken B. Correa P. A. Schnoor J. L. (2010). Phytoremediation of polychlorinated biphenyls: new trends and promises. Environ. Sci. Technol. 44, 2767–2776. doi: 10.1021/es902514d 20384372 PMC3025541

[B86] van Dam N. M. Bouwmeester H. J. (2016). Metabolomics in the rhizosphere: Tapping into belowground chemical communication. Trends Plant Sci. 21, 256–265. doi: 10.1016/j.tplants.2016.01.008 26832948

[B87] Vergani L. Mapelli F. Folkmanova M. Papik J. Jansa J. Uhlik O.. (2022). DNA stable isotope probing on soil treated by plant biostimulation and flooding revealed the bacterial communities involved in PCB degradation. Sci. Rep. 12, 19232. doi: 10.1038/s41598-022-23728-2 36357494 PMC9649793

[B88] Vergani L. Mapelli F. Marasco R. Crotti E. Fusi M. Di Guardo A.. (2017b). Bacteria associated to plants naturally selected in a historical PCB polluted soil show potential to sustain natural attenuation. Front. Microbiol. 8. doi: 10.3389/fmicb.2017.01385 PMC5524726 28790991

[B89] Vergani L. Mapelli F. Suman J. Cajthaml T. Uhlik O. Borin S. (2019). Novel PCB-degrading *Rhodococcus* strains able to promote plant growth for assisted rhizoremediation of historically polluted soils. PloS One 14, e0221253. doi: 10.1371/journal.pone.0221253 31437185 PMC6705854

[B90] Vergani L. Mapelli F. Zanardini E. Terzaghi E. Di Guardo A. Morosini C.. (2017a). Phyto-rhizoremediation of polychlorinated biphenyl contaminated soils: An outlook on plant-microbe beneficial interactions. Sci. Total Environ. 575, 1395–1406. doi: 10.1016/j.scitotenv.2016.09.218 27717569

[B91] Voges M.J.E.E.E. Bai Y. Schulze-Lefert P. Sattely E. S. (2019). Plant-derived coumarins shape the composition of an *Arabidopsis* synthetic root microbiome. PNAS 116, 12558–12565. doi: 10.1073/pnas.1820691116 31152139 PMC6589675

[B92] Wang C. Sun Y. Ruan H. Yang J. (2021). Toxic effects of 2,4,4′-trichlorobiphenyl (PCB-28) on growth, photosynthesis characteristics and antioxidant defense system of *Lemna minor* L. Plant Physiol. Biochem. 166, 505–511. doi: 10.1016/j.plaphy.2021.06.031 34166977

[B93] Wang Z. Teng Y. Wang X. Xu Y. Li R. Hu W.. (2023). Removal of cadmium and polychlorinated biphenyls by clover and the associated microbial community in a long-term co-contaminated soil. Sci. Total Environ. 871, 161983. doi: 10.1016/j.scitotenv.2023.161983 36740062

[B94] Williams A. Langridge H. Straathof A. L. Fox G. Muhammadali H. Hollywood K. A.. (2021). Comparing root exudate collection techniques: an improved hybrid method. Soil Biol. Biochem. 161, 108391. doi: 10.1016/j.soilbio.2021.108391 34602656 PMC8444088

[B95] Wrzaczek M. Brosché M. Kollist H. Kangasjärvi J. (2009). *Arabidopsis* GRI is involved in the regulation of cell death induced by extracellular ROS. PNAS 106, 5412–5417. doi: 10.1073/pnas.0808980106 19279211 PMC2664059

[B96] Xiang Y. Xing Z. Liu J. Qin W. Huang X. (2020). Recent advances in the biodegradation of polychlorinated biphenyls. World J. Microbiol. Biotechnol. 36, 145. doi: 10.1007/s11274-020-02922-2 32862310

[B97] Yaghoubi Khanghahi M. Strafella S. Allegretta I. Crecchio C. (2021). Isolation of bacteria with potential plant-promoting traits and optimization of their growth conditions. Curr. Microbiol. 78, 464–478. doi: 10.1007/s00284-020-02303-w 33354746 PMC7864805

[B98] Yang L. Ding W. Xu Y. Wu D. Li S. Chen J.. (2016). New insights into the antibacterial activity of hydroxycoumarins against *Ralstonia solanacearum* . Molecules 21, 468. doi: 10.3390/molecules21040468 27070570 PMC6273506

[B99] Yang L. Li S. Qin X. Jiang G. Chen J. Li B.. (2017). Exposure to umbelliferone reduces *Ralstonia solanacearum* biofilm formation, transcription of type III secretion system regulators and effectors and virulence on tobacco. Front. Microbiol. 8. doi: 10.3389/fmicb.2017.01234 PMC5492427 28713361

[B100] Yi H.-S. Yang J. W. Ghim S.-Y. Ryu C.-M. (2011). A cry for help from leaf to root. Plant Signal Behav. 6, 1192–1194. doi: 10.4161/psb.6.8.15780 21822064 PMC3260719

[B101] Yoshioka S. Newell P. D. (2016). Disruption of *de novo* purine biosynthesis in *Pseudomonas fluorescens* Pf0-1 leads to reduced biofilm formation and a reduction in cell size of surface-attached but not planktonic cells. PeerJ 4, e1543. doi: 10.7717/peerj.1543 26788425 PMC4715448

[B102] Zamcho F. Newborn A. Karamat A. Tehrani R. Pleshko N. Van Aken B. (2021). Effects of polychlorinated biphenyls on lignin biosynthesis in *Arabidopsis thaliana.* ACS agric. Sci. Technol. 1, 202–210. doi: 10.1021/acsagscitech.1c00022

[B103] Zhalnina K. Louie K. B. Hao Z. Mansoori N. da Rocha U. N. Shi S.. (2018). Dynamic root exudate chemistry and microbial substrate preferences drive patterns in rhizosphere microbial community assembly. Nat. Microbiol. 3, 470–480. doi: 10.1038/s41564-018-0129-3 29556109

[B104] Zubrova A. Michalikova K. Semerad J. Strejcek M. Cajthaml T. Suman J.. (2021). Biphenyl 2,3-Dioxygenase in *Pseudomonas alcaliphila* JAB1 is both induced by phenolics and monoterpenes and involved in their transformation. Front. Microbiol. 12. doi: 10.3389/fmicb.2021.657311 PMC8119895 33995321

