# Supplementary material for: Polychlorinated biphenyls modify Arabidopsis root exudation pattern to accommodate degrading bacteria, showing strain and functional trait specificity
Source: Front Plant Sci. 2024 Jul 5;15:1429096. doi: 10.3389/fpls.2024.1429096 (PMC11258928; doi:10.3389/fpls.2024.1429096)
Supplement: Supplementary file 1 [file DataSheet_1.pdf]

SUPPLEMENTARY MATERIALS

Polychlorinated biphenyls modify *Arabidopsis* root exudation pattern to accommodate degrading bacteria, showing strain and functional trait specificity.

Eleonora Rolli[1], Elisa Ghitti[1], Francesca Mapelli[1], Sara Borin[1]

[1]Department of Food, Environmental and Nutritional Sciences (DeFENS), University of Milan, Milan, Italy.

*** Correspondence.** Mail address: sara.borin@unimi.it. Phone number: +39-0250319118

SUPPLEMENTARY TABLES

Supplementary Table 1. List of the 200 annotated features detected by LC-MS in the metabolomics analysis. Among them, the five compounds in bold are those with a statistically different relative abundance between the REs of mock-treated and PCB-18 exposed plants. Peak_ID: code for the detected peak. PP, PN: metabolic features detected in polar positive and negative ionization modes, respectively. mz_mean: it corresponds to the average value of all the detected m/z in the various samples for a specific feature. The remaining columns reported the normalized intensity values for the four biological replicates of REs collected at T2 from the mock treatment (M1, M2, M3, M4) and for the PCB-18 exposed plants (P1, P2, P3, P4). NA stands for not available

Peak_ID	Compound_Name	mz_mean	T2M1	T2M2	T2M3	T2M4	T2P1	T2P2	T2P3	T2P4
PP_017474	**Scopoletin**	193.0495163	22.40548429188285	22.74971314922418	22.33071504823189	21.955572580116453	18.796521305784047	18.71746892435344	18.112459869880855	17.612234991145264
PP_022099	**L-Arginyl-L-valine**	274.1873564	17.02723648740348	16.9767319209274	16.873864612982047	17.326771791638954	17.66421114946924	18.047789581710852	17.812446832819532	17.932537734788514
PP_016782	**N-(2-Hydroxyethyl)-beta-alanine**	134.0811833	19.60800750184744	19.73900263133906	19.641651366820394	19.62357789670497	19.217264328266346	19.14683215671486	18.983859248250194	19.26759824910029
PP_016186	**Hypoxanthine**	137.0458586	18.11036605753233	17.672003997143747	17.03028994839212	18.12611963053309	19.23425610757446	19.17162395364399	19.573358726146854	20.174099129237444
PP_022339	**L-Seryl-L-phenylalanine**	253.1182787	18.895622614118714	18.55653599076242	18.70878079538074	19.124565621917384	19.37741814014738	19.741248066416542	19.699431812402615	19.824357471606206
PP_023128	L-Tyrosyl-L-tyrosine	345.1442831	18.018265390772385	17.94297260556882	17.799484005620016	17.850357786098144	17.449656479065045	17.33289320893788	17.55984757474703	17.626357852760833
PP_005075	4-hydroxy-4-[(E)-3-hydroxybut-1-enyl]-3,5,5-trimethylcyclohex-2-en-1-one	207.1378556	19.595255660010427	19.781012254224322	20.200291592810157	20.09179950598771	20.888980272316108	20.99643211128595	20.393655449547893	20.762506131776895
PP_016018	Glycyl-L-proline	173.0920503	18.57935277062292	18.37545948857944	18.71639413899008	18.940651975905627	19.366674391726118	19.380647072009847	19.226372207273258	19.491338371277966
PP_021646	L-Methionyl-L-valine	249.12668	16.54710540004083	16.316405287042162	16.280462685894967	16.376108427468402	16.7100934103752	16.780821676493435	17.123434381523797	16.901432607729507
PP_017079	Nicotinamide	123.0554833	20.400157099311794	20.29272042382496	20.31853177691288	20.682641463016413	20.924639281906412	21.05364155891374	20.913508877371218	20.956918478372835
PP_014339	2-Picolinic acid	124.0394873	20.868906934537364	20.708919686150487	20.48872865075274	21.673623516150865	22.100519583209664	22.490637423668318	22.263835966237657	22.64474175240821
PP_019167	Uracil	113.0349087	18.993391761720428	18.734554171832297	17.959102003983784	18.0271392747597	20.05215939065413	19.50118214905818	19.891915687167163	19.104823952345146
PP_016274	Kaempferol	287.05469	21.12293602609946	20.712475782728283	21.24802483423795	21.52831531443854	22.084521653147664	22.489052673376744	21.524761686609672	22.129467920841506
PP_018571	Adenine	136.0617979	24.34135810946341	23.67818037909157	23.737911498155643	24.922561220577432	25.49352450235269	25.75393129303584	25.587209251524943	25.73342866106671

PP_020254	L-Alanyl-L-threonine | L-Threonyl-L-alanine	191.1023333	20.71108717401154	20.79306981988077	20.699958721557977	20.900225594778576	21.773021660928993	21.867583412182125	21.12351584531683	21.519422697380563
PP_020875	Glycyl-L-threonine | L-Seryl-L-alanine | L-Threonylglycine	177.0868723	21.335167882508504	21.153646654999623	20.73423247276076	20.623155438548416	20.44326936418982	19.99629003968459	20.07476212597023	20.313319288240614
PP_022210	L-Glutaminyl-L-leucine	260.1606515	20.10349656398597	19.544028646884847	19.95747901703359	20.600592612765123	20.6202797957259	21.150027380400562	20.93209000933685	21.41071943857476
PP_022306	L-Seryl-L-leucine	219.1338438	20.52775379234216	19.964074297946837	20.233558826757562	20.967281149293424	21.034767400054626	21.42636150969686	21.333618350361018	21.642566045892863
PP_022639	L-Valyl-L-lysine	246.1809491	19.789240695155943	19.929400012378498	20.0875172272094	20.00615620819151	20.467775680447286	20.173446573573877	20.202811356989173	20.246706970467976
PP_018248	Deoxyadenosine	252.1089644	22.54021461267461	21.93306531010247	22.413901364603397	23.038831935979676	23.4274145163957	23.710656480020727	23.01851788948682	23.69837296496247
PP_022479	L-Threonyl-L-leucine	233.1492611	20.208167664165035	19.58165481044163	20.015227189238967	20.783670509489365	20.85399403344063	21.36073994089506	21.125781095141825	21.501079590557865
PP_011614	Genistein 7-O-beta-D-glucoside	433.1115143	22.31408725694412	21.833561085231487	22.444284700638573	22.751766529226856	23.263115436050516	23.679662426507033	22.75445449953067	23.25357355780802
PP_022855	L-Tryptophyl-L-glutamic acid	334.1395933	18.061566326414123	17.940170192372545	17.945318088904923	18.388664805112523	18.360045459501983	18.637495174271073	18.46038390054939	18.662247648643792
PP_018160	Guanine	152.0566656	23.429908676909037	22.437936170050726	22.638666805419295	23.94341857669974	24.516643415547243	24.807167497932223	24.337505125565432	24.902821761739002
PP_022390	L-Seryl-L-tryptophan	292.1291581	18.303196568128858	18.059640203274725	18.149523863905884	18.520906374512368	18.4427208105509	18.803062144435902	18.75480596734355	18.902737226761236
PP_014251	Deoxyguanosine	268.1040728	20.209257013154886	19.676301461720342	19.85422161325518	20.5715730507989	20.89307707007295	21.147099317056302	20.496509559647528	21.127802302402092
PP_022349	L-Seryl-L-isoleucine	219.1336714	18.412611192709466	17.82743574791787	18.09309770952382	18.901441446590166	18.900182837097066	19.356269266114136	19.26171618736046	19.522338236780637
PP_016050	Guanosine	284.0988601	21.700660685695123	21.149311739081615	21.397921610000836	22.74262399450218	23.015273987147904	23.570545864814605	22.789754910014008	23.396598248888342
PP_021359	L-Lysyl-L-valine	246.1809899	17.820565418067776	17.217011035117455	17.411494437175566	18.12945911643141	18.099060097540992	18.574722861515678	18.479946005317224	18.81744276445619
PP_021509	L-Leucyl-L-valine	231.1700862	19.651240931556217	19.201114050919884	19.514806500021418	19.98973541867687	19.89527646493906	20.36864393284131	20.368985955828673	20.59620340803598
PP_021751	L-Asparaginyl-L-Isoleucine	246.1448361	17.795500678217547	17.215286513232048	17.481720610946148	18.137491516183978	18.07222159721205	18.612530182029023	18.497664753369076	18.776317361336776
PP_021452	L-Leucyl-L-phenylalanine	279.1703423	19.65613502698934	19.14550929494578	19.35739306711537	19.69002355621454	19.854763397961555	19.96805220983374	20.280703948622786	20.64630116852243
PP_016686	L-Methionine	150.058149	24.06922897762936	23.90097420175199	24.00945610723742	24.246246028080808	24.364046530389743	24.353125484765194	24.20497942342321	24.45599005280463
PP_022569	L-Threonyl-L-isoleucine	233.1491969	18.011112574433575	17.495089890931368	17.861153561232495	18.883672479135388	18.726939885191726	19.4538593903075	19.07184726592027	19.635822609357486
PP_002492	(3R,5R)-1-[(E)-3-(3,4-dihydroxyphenyl)prop-2-enoyl]oxy-3,4,5-trihydroxycyclohexane-1-carboxylic acid | (3R,5R)-4-[(E)-3-(3,4-dihydroxyphenyl)prop-2-enoyl]oxy-1,3,5-trihydroxycyclohexane-1-carboxylic acid	355.1021628	18.586887421673207	19.073056294104887	18.451859051972693	18.18124448046612	17.399077610477097	17.449640360107683	18.345478883553003	17.257838982683523
PP_022679	L-Valyl-L-glutamic acid	247.1285612	20.778536788257462	20.357715219505092	20.676548538713224	21.05507982829861	21.095844473005947	21.547299833176424	21.078440765844398	21.639049186398676
PP_014419	3,6-Anhydrogalactose	180.0865175	26.88034014275905	26.825771759144093	26.1255665142993	26.77855737872414	24.735583705489272	24.36265967956339	24.26722024701252	26.219143957898638
PP_015914	D-Galactose | D-Mannose | muco-Inositol | myo-Inositol | D-Tagatose	219.0262187	29.04921576894826	28.984439030020557	28.415599517775807	28.83955477325453	27.492421856949335	27.055607712418464	26.972130636697234	28.482317289841067
PP_020448	L-Aspartyl-L-tyrosine	297.1080578	19.53500762624472	19.28035166883295	19.458825111610427	19.62577304384802	18.82026623564383	19.21352433048501	19.058427802267484	19.270409401466043

PP_021982	L-Prolyl-L-alanine	187.1076329	18.566299363495734	18.07558360404767	18.375179464014256	18.927523357266224	18.933990263339	19.501743904190114	18.923359406179618	19.52845983403797
PP_017104	N-Methyl-L-glutamic acid	162.0759789	21.476771909625384	21.465669526494505	20.79738526357619	21.487543215849183	19.334426060689974	19.10817219330654	18.957833227516936	20.926715123310405
PP_020186	L-Alanyl-L-isoleucine	203.139021	17.883013131875032	NA	17.652788966672624	18.14435950903393	18.176489717041722	18.58829373934542	18.547945088051197	18.735357692596175
PP_022400	L-Seryl-L-valine	205.1181095	18.51616938915585	17.875113377400325	18.240940363208097	19.146272823717077	19.124553001359978	19.572938874852824	NA	19.751671539287692
PP_022649	L-Valyl-L-methionine	249.1269137	17.552098678383203	17.455961278490804	17.34924666786686	17.489173734259815	17.656019774695018	17.989499832962064	18.141054375730928	18.359166160994807
PP_023002	L-Tyrosyl-L-phenylalanine	329.1493139	18.165932542214332	18.00640278760374	17.99255115278717	18.14432465272781	18.206351445137788	18.42489617976851	18.614328820644158	18.333399972624868
PP_001844	3-[4,5-dihydroxy-3-(3,4,5-trihydroxy-6-methyloxan-2-yl)oxy-6-[(3,4,5-trihydroxy-6-methyloxan-2-yl)oxymethyl]oxan-2-yl]oxy-5,7-dihydroxy-2-(4-hydroxyphenyl)chromen-4-one	741.2233628	18.11272965381611	18.325665591940435	18.299123035707716	18.11700382704303	17.58324440330465	17.88532697858409	18.08104646900816	17.628602172954466
PP_016008	Glycyl-L-phenylalanine	223.1075012	19.456007417417872	19.08073686914283	19.32264996621974	19.68762001703156	19.61704949326128	19.941560617571614	19.784523025202255	20.04082882199412
PP_023048	L-Tyrosyl-L-valine	281.1495264	19.30362469269398	18.944337454268368	19.15175982067403	19.419089905146762	19.322163623099275	19.711140962325945	19.673202089750095	19.877962574178472
PP_014948	allo-Inositol | alpha-D-Talose	203.0523966	24.900018613704106	24.80784096142299	24.364877320308025	24.701877728712773	23.34959993356155	22.973876005110018	22.844608081692762	24.431145277008998
PP_020136	L-Alanyl-L-aspartic acid | L-Aspartyl-L-alanine | L-Glutamylglycine | Glycyl-L-glutamic acid	205.0816639	20.28757261169725	20.14001965466775	19.701015348285985	19.822944202454998	19.437508265386228	18.91556431494779	19.170560814940828	19.785576987188527
PP_017087	Nicotinic acid	124.0394856	24.383784897563867	24.40447596104304	24.38064548682491	24.35355284516917	24.867609945429948	24.814436710456793	24.553472450029115	24.543426125812136
PP_015396	D-Trehalose | D-Maltose | Galactinol | Turanose	381.0790035	22.39212793459196	22.412259973219204	21.614858092562834	22.54533593746865	20.732829887007615	20.52406340022223	20.171669150225625	22.046834249948155
PP_015382	D-Raffinose	522.2027667	21.288643068432723	21.48979750238265	20.212896492532536	20.9578913097364	19.435336566567898	18.96682929644702	20.15766026035254	20.524690916806286
PP_016574	L-Ornithine	133.0974201	19.1840520499009	19.34724071480908	19.131932299918386	18.22726338610897	18.124767535822475	18.63136181526636	18.03691409182039	17.710342335912333
PP_022734	L-Valylglycine	175.107694	18.97444259911367	18.609344691634657	18.567020229834327	18.936039759029036	18.853757281891884	19.272381472380243	19.499304806125203	19.55486985128006
PP_023107	L-Tyrosyl-L-Asparagine	296.1239399	17.097229722757675	17.136330312785574	16.92977851741178	17.49991594028229	17.33470974348605	17.629826170951414	17.590572431092582	17.668822673648503
PP_015157	Betaine	118.0864935	23.64851393309112	23.515096558673118	23.50559242901661	23.292748222750923	24.465940932944893	24.33948312786264	24.551932811092588	23.5697894496141
PP_022659	L-Valyl-L-threonine	219.1337242	19.8886823149565	19.446752161539603	NA	19.878751440517394	NA	20.247313314139678	20.193302761649498	20.706189926872483
PP_021197	L-Isoleucyl-L-tryptophan	318.1811356	18.544311710685214	18.42422370935301	18.513654355363858	18.693514182013	18.569113900926357	18.824797183836907	18.834208693251234	18.811794866422392
PP_022716	L-Valyl-L-alanine	189.123356	19.19553081054588	18.825340812248598	18.943822568701425	19.306693655973618	19.278882720754304	19.67763706744639	19.72812811678029	20.34746782146691
PP_022752	L-Valyl-L-asparagine	232.1290004	19.921512644772545	19.456807592756263	19.61418141664789	19.89110876509742	20.110455272093514	20.06975000044757	19.902857910658202	20.481822091825656
PP_022014	L-Prolyl-L-threonine	217.118078	22.806738601160777	22.579717597164148	23.432340672129232	22.759070979078718	22.277020300901153	22.002304445898773	22.69624019862026	22.200905787086203
PP_022993	L-Tyrosylglycine	239.1021269	18.47351550830037	18.225551083153416	18.36848088400031	18.835137530154583	18.53163655981468	18.99283325380845	19.152341790713596	19.222804334682476
PP_022609	L-Threonyl-L-tryptophan	306.1448135	18.051373588778933	17.78486484875305	18.01139652974862	18.315587421410896	18.096797497745936	18.616247293597404	18.436996136666156	18.648375138063294
PP_017796	L-Tyrosine	182.0812007	NA	NA	25.836258001826565	25.881066780849586	NA	25.586495885445533	NA	25.584932776080116
PP_015237	Choline	104.1073676	21.270213186890818	21.58964977511711	21.63212192296844	21.16305934798352	22.395007146689345	22.415924055361717	23.014529975907365	21.495989664440323

PP_021947	L-Prolylglycine	173.0919074	18.003111591831942	17.568891314327	17.740090365748756	18.693034274675927	18.576388388324006	18.792532955833906	18.38156407369676	19.02102604209805
PP_020426	L-Aspartyl-L-valine	233.1128208	19.236441699669594	18.932214751968385	19.094704005867428	19.340834579635587	19.392200011178215	19.690441376987106	19.228574463633237	19.830091164836293
PP_015959	D-Glucosaminic acid	196.08154	19.386462472142757	19.159013930136123	NA	19.092710693516928	17.071828939517054	16.790272123945538	NA	18.416471809294443
PP_016493	L-Glutamic acid	148.0603267	23.239552206706815	22.65315778087073	22.746426160818757	23.13072639859259	23.493432576154277	23.455801034560004	23.0888233309631	23.296501910368463
PP_021234	L-Isoleucyl-L-asparagine	246.1447213	17.944786398912665	17.60076953654753	17.807637928603192	18.122271916329602	18.01210071615157	18.52217154680393	18.293570435579475	19.08563324299889
PP_021928	L-Prolyl-L-isoleucine	229.154461	18.42287782743315	18.223766055982896	18.58036899163762	18.76334530466388	18.644377381913777	19.084014213723922	18.958362964087282	19.732814984972034
PP_022707	L-Valyl-L-aspartic acid	233.1128858	20.176207244376617	19.793331825933734	19.806024047149993	20.142569545266838	20.43850355111329	20.61311048799384	20.007448911412407	20.952911838336565
PP_020360	L-Aspartyl-L-leucine	247.128546	21.641300328744865	21.386972862833222	21.685169682836072	22.02672267140592	21.892644717972324	22.378654176598747	21.834204834707997	22.395604211774973
PP_017055	N-gamma-Ethyl-L-glutamine	175.1077101	18.86893405555578	18.448930947631464	19.04443414385654	19.558224502848493	19.26167487320094	19.63693786805665	19.48629100223904	19.9658772678694
PP_020513	L-Glutamyl-L-leucine	261.1444987	21.56406387507252	21.250743107231248	21.48822163728448	21.851827183451444	21.716836622296483	22.21786956612125	21.68518936364173	22.24992214648622
PP_023030	L-Tyrosyl-L-alanine	253.1183498	18.252415969502305	18.132790890085783	18.17085906163548	18.41079499079705	18.238875844747266	18.473091338381973	18.674837071179013	18.705264512619923
PP_015374	D-Melezitose	522.2026875	21.288643068432723	21.48979750238265	20.212896492532536	20.9578913097364	19.435336566567898	17.826728465378736	20.15766026035254	20.524690916806286
PP_021462	L-Leucyl-L-glutamic acid	261.1445032	21.548656369449116	21.378416050652014	21.536795010182857	21.682219233242986	21.583557062078974	22.069099635171078	21.7376013771035	22.22025461713021
PP_022789	L-Valyl-L-arginine	274.1870597	18.096581336670607	17.804219270592718	17.56097461907799	18.092824228973104	18.124923999664286	18.497777785934122	18.810521428722772	19.875161385221663
PP_016421	L-Citrulline	176.1028832	24.65170741038668	24.52576147698977	24.492345326063294	24.669139823988527	24.591238921640883	24.42525054856952	24.347778413045976	24.413182032675838
PP_021161	L-Isoleucyl-L-glutamic acid	261.1445474	20.883304803406602	20.63715373918228	20.970716089740236	21.168860609561875	21.08422123422461	21.637081638280048	21.01019479818922	21.562938776774494
PP_022742	L-Valyl-L-glutamine	246.1445987	19.49410945551113	19.166467143469998	19.054954012120792	19.374642598294642	19.407904629156914	19.443400905088367	19.547474471111382	19.87917039713007
PP_022864	L-Tryptophyl-L-aspartic acid	320.1239785	17.49095269643774	17.565883029180622	17.386704535599407	17.793215913973157	17.614019614435723	18.023277880370888	17.70171256893974	18.0719179512949
PP_015582	L-Aspartic acid	134.0448428	18.042134938399297	18.216011632096674	17.75099478480682	NA	NA	16.459575701634073	17.029157792779344	NA
PP_023069	L-Tyrosyl-L-serine	269.1130294	17.571974789113394	17.501891595865924	17.414255798375592	17.608991115551703	17.50098188586893	17.730848221914147	17.90822254800495	18.02054052865649
PP_023020	L-Tyrosyl-L-aspartic acid	297.1078861	19.05531951086363	18.7193955060126	18.889759425961206	19.27375918177561	19.072603681429907	19.481048489416125	19.130113012109977	19.606701598707303
PP_016598	L-Lysine	147.1127847	19.894302659919976	20.272822254786277	19.59057791316836	19.64790456195126	19.854367732538194	19.17296947230598	19.50953727216098	19.179124275128583
PP_020646	L-Phenylalanyl-L-glutamic acid	295.1289191	19.998047708459026	19.865163867538772	20.04512239148163	20.1040893675658	19.978580595963	20.36396361216805	20.155261715048955	20.445430173777666
PP_013962	(S)-(+)-2-Amino-2-methyl-3-hydroxypropanoic acid | Allothreonine | beta-Homoserine | gamma-Amino-beta-hydroxybutyric acid | L-Homoserine | L-Threonine	120.0657236	23.183960913964665	22.981755050922413	23.111072453874044	23.02959273323617	23.14343911350294	22.736244600349846	22.584977405807237	22.818848366067552
PP_020524	L-Glutamyl-L-methionine	279.1008757	17.763030773372286	17.683570362091572	17.781007450053032	18.002067812650534	17.85016852053869	18.229823343013482	17.87531739960898	18.247816281639192
PP_020636	L-Phenylalanyl-L-aspartatic acid	281.1134108	19.183674146403852	19.015943116280972	19.249115768047737	19.405635941640664	19.245165037579667	19.743838409765093	19.332508648743445	19.89289554983811
PP_020906	Glycyl-L-tyrosine	239.1022752	18.63931244240666	18.341249508934254	18.517500235509672	18.792218368691422	17.990425324911946	18.370964048928265	18.39011436361919	18.48223680840405
PP_023011	L-Tyrosyl-L-glutamic acid	311.1235414	19.861956211993068	19.576172458421173	19.842743180731656	20.06766674846228	19.86396995881347	20.307098503119963	19.959471382011834	20.336075004766002
PP_020655	L-Phenylalanyl-L-phenylalanine	313.1541458	18.107789200646963	18.066288943461554	17.95940204238948	18.18201601537938	18.218303075081984	18.231708698048106	18.688113683815505	19.326552375011524

PP_021094	L-Isoleucyl-L-alanine	203.1389259	19.391465972106662	19.1650247377713	19.34340261705982	19.46139151439007	19.407697212174547	19.738794879691355	19.676017313914205	20.62216981742743
PP_018128	Xanthine	153.0406996	20.91009371100127	21.005242812310886	20.407822703012013	20.85119620858192	20.745025944964382	20.36409510315375	20.5422440666977	20.43287012640862
PP_022973	L-Tyrosyl-L-isoleucine	295.1652866	18.902672465386107	18.63211482101171	18.760625748253428	18.887039100788375	18.71008331789341	19.02925217302203	19.144202649024066	19.088865860974465
PP_015422	3-Aminoisobutyric acid | N,N-Dimethylglycine | (R)-2-Aminobutanoic acid	104.0709982	25.494254695249147	25.04212424828518	24.971207475783224	25.96951429441894	25.705632387361415	25.703642815933716	25.643588009501244	26.26258184492161
PP_020726	L-Phenylalanyl-L-asparagine	280.1292653	17.27658789839825	17.224548160305932	17.220850633234377	17.37848114231423	17.32969124297091	17.665648197101643	17.75675827527008	18.84338438058346
PP_020617	L-Glutamyl-L-tyrosine	311.1237808	19.421292344292624	19.284812806538127	19.406240304901356	19.544975749065145	18.80253845268592	19.243087960067744	18.937353703431537	19.50153205683816
PP_015318	Cytidine	244.0923947	23.809376708638876	23.48870573111699	23.69797535218012	23.96471622110587	24.109084901677548	24.385711224855406	23.604508437341416	24.119652148413177
PP_021121	L-Isoleucyl-L-lysine	260.1968324	18.673505770705905	18.57995669839254	18.54313757694867	18.729909460770394	18.638693979683275	18.893283753061745	19.389154456597332	20.307875624695566
PP_022780	L-Valyl-L-serine	205.1180811	18.74083482614526	18.32783768151511	18.25441508002101	18.423637092028518	18.421871699470206	18.669265712909866	18.662963854102	18.864143817110048
PP_020627	L-Phenylalanyl-L-alanine	237.1230514	18.21633852728154	18.158200401132063	18.177692042079045	18.28639531932448	18.288325394685337	18.525926020782865	18.715335312167095	20.36470536375136
PP_020314	L-Aspartyl-L-phenylalanine	281.1132063	20.57433112809745	20.40963643363459	20.646532342816734	20.688181144183183	20.61950446608572	20.92501106691288	20.5332831490896	21.003403302942033
PP_020763	L-Phenylalanyl-L-threonine	267.1340978	18.82842164222088	18.65902728759254	18.748933141725598	18.78982335204021	18.711586319753515	18.934880079059067	19.157778664678403	19.99314294826744
PP_022253	L-Glutaminyl-L-tyrosine	310.1396116	17.593427598236804	17.341075731081744	17.438913652786095	18.027004246331103	17.445507971305705	18.03964199840991	17.901668292150156	18.348823229016258
PP_021529	L-Leucyl-L-asparagine	246.1446386	18.681735982169165	18.647637599806355	18.471214773888875	18.652795967582694	18.44807596463427	18.916929095144766	19.04039943035261	19.90496824629866
PP_015927	gamma-Valerolactam	100.0761292	18.784660403279	19.150937232895423	19.134366153192264	18.88343085290352	19.38109508113716	19.317600305253407	18.816436835996463	19.26648918108471
PP_020283	L-Alanyl-L-tyrosine | L-Phenylalanyl-L-serine	253.118347	17.50846022203516	17.152344266680302	17.065395096623003	17.34946266022597	16.527278450778272	16.998964981189392	17.203414920329458	17.29858171273781
PP_020735	L-Phenylalanyl-L-glutamine	294.1448105	18.0803074820617	17.963200901458812	17.908246007509902	17.89945723772608	17.87649296539759	18.124757440798344	18.314556692663537	19.195030911212722
PP_021481	L-Leucyl-L-alanine	203.1389472	19.742789675476782	19.638969670104885	19.570274504489287	19.719631134959236	19.53022723890187	19.856686915605994	20.161141726666887	21.263244553327034
PP_022539	L-Threonyl-L-tyrosine	283.128388	17.803052681813675	NA	17.56241386526774	18.110422135900517	17.668656498842903	18.340654232841597	18.073984621365497	18.389127141363627
PP_023088	L-Tyrosyl-L-proline	279.1341175	20.425708671630797	20.392205252971298	20.45920646014343	20.780796050676745	20.527162932487773	20.878830752354748	20.48792735046126	20.852389300176352
PP_021170	L-Isoleucyl-L-arginine	288.2029357	NA	17.059819731617495	17.11873834166337	17.55040861410204	NA	17.903728890117698	NA	19.092302924351287
PP_017852	Urocanic acid	139.0502101	18.451782602129967	19.19830114846813	19.533428597821782	19.09889052558918	20.860627052446805	19.402002353199524	18.381344384452287	20.840349971794726
PP_021964	L-Prolyl-L-glutamic acid	245.1129399	19.735594034300124	19.416991274502696	17.337299586983555	18.033030397065026	19.813582368630538	20.167001546698618	19.56119843036924	18.24369466432139
PP_016582	L-Tryptophan	205.096889	27.246798440124106	27.5998367210123	27.511791999091894	27.501239591999564	27.90410417811512	27.659895024161656	27.6798735153184	27.299248979159444
PP_021110	L-Isoleucyl-L-histidine | L-Leucyl-L-histidine	269.1608203	16.95554774933441	16.66963250183935	16.960047196639138	17.0163621634505	17.18212273615043	17.0783593228228	17.009478745888064	19.959312886549235
PP_021472	L-Leucyl-L-aspartic acid	247.1284861	NA	16.29641438743592	19.96562789029726	20.31828241335956	NA	20.43270500339691	NA	20.786090115675503
PP_023098	L-Tyrosyl-L-glutamine	310.1392003	17.636858235305986	17.0195364351638	17.4481324414037	17.238563346446607	17.79247894069762	17.245794076038834	17.52460292788525	17.506385069944024
PP_020664	L-Phenylalanylglycine	223.107484	18.65335243125208	18.620567081150313	18.33434312748961	18.47858399450532	18.36857892864948	18.476188475531703	18.91454280151104	19.53492976042596
PP_020417	L-Aspartyl-L-threonine | L-Glutamyl-L-Serine | L-Seryl-L-glutamic acid | L-Threonyl-L-aspartic acid	235.0915405	24.571474971246566	24.84218515867228	24.441192464706532	24.97876020197212	24.272451528197447	24.771060363681666	24.53873403649975	24.618623153079742

PP_014523	3-Ureidopropanoic acid	133.0608129	19.271864207087894	19.325994906703876	19.218643640133134	19.23323815165567	19.748102743865008	19.661200514536564	19.382510045111683	18.98121885163232
PP_015831	Ethylenediaminetetraacetic acid	293.097299	22.31188188102181	21.865597736995397	21.335808487747574	21.615643106139217	21.560587063672873	20.718766973944913	22.241591611224873	21.191471334962735
PP_021207	L-Isoleucyl-L-glutamine	260.1603462	19.034503456611986	18.75860385545385	18.418585606299196	18.601756282472483	18.865914466144808	18.727255734529653	18.613357834307436	19.393342271831653
PP_021444	L-Leucylglycine	189.123385	19.76251180898212	19.59532851576121	19.457651407125365	19.682824890848597	19.353097276813674	19.541344330357767	20.167643549322687	20.470511921884793
PP_016557	L-Methionine methylsulfonium	164.0740889	20.78191035316208	20.69604381885305	20.25987541213727	20.23523594278313	20.605340250872135	20.128704252956208	20.51996252373815	19.963919128797293
PP_020545	L-Glutamyl-L-glutamine	276.1187353	18.793273077199796	NA	NA	18.815448969167566	18.699711386241166	NA	18.902884400894134	19.408014548068067
PP_020895	Glycyl-L-tryptophan	262.1188812	18.241303561899592	18.048561373848766	NA	18.46735081448758	18.284720194973918	NA	NA	18.501802205342013
PP_020372	L-Aspartyl-L-methionine	265.0853838	17.092044783031582	16.533664326714817	16.754104577609123	17.13268045682941	16.794999609194814	17.257710099937974	16.76312157295051	17.360855655298696
PP_017589	Sucrose	381.0788999	24.294331456024253	24.094876647165275	22.76271090254962	23.24725709604792	23.748383998441987	22.519304868419322	26.36589296508326	24.517564511037623
PP_020885	Glycyl-L-valine	175.1077016	17.957283455588414	16.795202594832023	16.81764573949385	17.820328570432146	17.86660883735756	18.038404628941894	16.56016861081948	18.46394338492975
PP_021151	L-Isoleucyl-L-leucine	245.1858287	19.02031263360589	18.928336199450115	18.816158676101928	18.82021013203148	18.67868609638469	18.8058414673715	19.319954310686942	19.31131150658815
PP_021601	L-Methionyl-L-glutamic acid	279.1006698	NA	NA	16.995700913970378	16.704092763401494	17.02068701360999	17.176075729612624	16.5251996170952	17.34105835214517
PP_020502	L-Glutamyl-L-isoleucine	261.1446669	19.084307538073254	18.88868380118082	19.136760867895763	19.229527653480428	18.960496936150378	19.38233272271214	18.974448200910043	19.433365382079536
PP_016373	L-Arginine	175.1188776	21.229503009950218	21.213321410860566	20.972517795109894	21.37918743613536	21.021356857094798	21.125547163016275	21.429154367139827	20.891016771057878
PP_020794	L-Phenylalanyl-L-tyrosine	329.1494218	17.630444890967194	17.44860030674729	17.464721226717845	17.65036461006276	17.063184391979497	17.159102651008908	17.518975808282164	20.27800471842524
PP_020437	L-Aspartyl-L-tryptophan	320.1240505	18.7452213100806	18.652575422593976	18.838622276430918	18.964572137170315	18.703349374380622	19.00210902877977	18.695683684443196	19.006501407569434
PP_018008	Nicotine	163.1229057	27.62805656833357	27.805577141959382	28.11085623138911	27.9289631962327	27.97013531151973	28.187844099207293	28.09872527512732	27.53663000453588
PP_020607	L-Glutamyl-L-tryptophan	334.139545	18.47109170024649	18.323551797747175	18.451504934415546	18.454255108479604	18.18002082459538	18.488616868124065	18.302103382357856	18.563181373190687
PP_013889	(+)-Methyl-(S)-3-hydroxyvalerate	133.0858574	21.384149772036455	21.61848408477225	21.79429012450948	21.719421828176653	21.33571963779916	21.91481831654144	21.856453265245886	21.661271420110054
PP_014667	5prime-Deoxy-5prime-(methylthio)adenosine	298.096683	19.315005791636143	19.56159747435543	19.675281713912767	19.808788250291425	19.04158162094982	19.775465607939655	19.92896648768017	19.993795301186374
PP_021826	L-Asparaginyl-L-tyrosine	296.1240345	17.51918318971803	17.073503534762796	17.32586758057542	17.688065861897407	17.096643100282783	17.58185521399147	17.390605622494515	17.845073451811253
PP_012829	6-Hydroxy-4,4,7a-trimethyl-5,6,7,7a-tetrahydro-1-benzofuran-2(4H)-one	197.1170044	18.477544843586116	18.408495604006774	21.074739917366895	20.08238426905768	18.606369063896587	19.8252833526414	19.10884601354233	19.600415667283144
PP_017330	L-Proline	116.0708031	24.534411062096286	24.223681813132618	24.527765661945644	24.615965711330947	24.674939519190918	24.353848537579374	24.42214197278477	24.308463607473794
PP_020206	L-Alanyl-L-leucine	203.1390445	19.88831814385114	19.421869645430213	19.56729139042135	20.093822440210605	20.205348207907925	20.551987945900553	16.8924242315014	20.533073030585186
PP_014571	4-Guanidinobutyric acid	146.0924124	22.374008924611523	22.350610656754196	22.1654087842267	22.189675279639896	22.315458620582202	22.08734564548871	22.171854197137712	22.43604913428708
PP_014932	Allantoic acid	177.061753	17.632498276654935	17.026728749142865	NA	NA	17.76133046908554	17.435955060928627	16.969937320619714	17.325384517221842
PP_015510	Norleucine	132.1019494	28.025758208011176	28.08808558246407	28.112641971617446	28.019904613247597	28.283023343773642	28.01330699637697	28.021750490802372	27.859585898202727
PP_015550	Pyroglutamic acid	130.0501532	NA	NA	23.25078791552344	23.498791637904976	NA	23.488271660075004	23.236849086618758	NA
PP_016114	L-Histidine	156.0768499	19.66331139850527	NA	19.299807227809485	19.216475895907045	19.735227109595467	19.56652792455258	NA	18.84452801523779
PP_016501	L-Glutamine | L-Alanylglycine | Glycyl-L-alanine	147.0763388	26.52901504753374	26.232618799731775	26.168644557611824	26.309380732113127	26.651506105580186	26.36199320352512	26.223999189363017	26.105499397794247

PP_016533	L-Isoleucine | L-Leucine	132.1019648	28.947136831970386	29.00863395006836	29.013612649464864	28.9253602415022	29.184154542565633	28.980154979029034	28.936552458986878	28.737328525420907
PP_016950	N-Acetylputrescine	131.1179613	21.20086508834281	20.13166482755976	20.433305267514882	19.898347485877448	19.946279492787387	19.918145518730956	20.67886143336765	21.098585374338573
PP_017015	N-epsilon-Acetyl-L-lysine	189.1233382	21.66513767110888	21.312328489175375	21.154784122466182	21.04899166826768	21.311318713891406	21.003636629369343	21.489282533349424	21.34111320370137
PP_017128	Norvaline | L-Valine	118.0864722	28.715271296402303	28.68196971532528	28.673247484869048	28.65616167640752	28.953406924333116	28.648582807909385	28.623060203703655	28.47608642757956
PP_020586	L-Glutamyl-L-threonine	249.1077149	NA	20.46856239680471	20.255645256167178	21.348764348211756	20.648246096083337	20.608221427209983	20.847818186402108	20.730883040524844
PP_022761	L-Valyl-L-tyrosine	281.1497243	18.093757977143678	17.92298501828402	18.096076834840254	18.212762344276808	17.49366002936594	18.049354026914394	18.083027375540773	18.9354470862296
PP_015462	beta-Hydroxynorvaline	134.0812777	NA	NA	NA	NA	NA	NA	NA	19.26759824910029
PP_015566	D-2-Aminoadipic acid	162.0758349	NA	NA	NA	18.470563555436033	NA	18.56780385616824	18.170986164439416	NA
PP_016558	L-Methionine sulfoxide	166.0531193	NA	NA	NA	NA	17.131977501615747	17.2834591827729	17.3015051260603	17.672411529337964
PP_016918	N-Acetyl-L-ornithine	175.1074122	19.950527420124203	19.72790384029447	NA	19.204666658502667	NA	19.71343350487162	NA	NA
PP_020245	L-Alanyl-L-serine	177.0871304	NA	21.436514325855853	NA	NA	NA	NA	20.587020238138777	20.820096359675023
PP_021973	L-Prolyl-L-aspartic acid	231.097156	NA	NA	NA	18.157006394826325	18.274032290160882	18.483485163749144	NA	18.585633764801415
PP_021179	L-Isoleucyl-L-serine	219.1336032	NA	NA	NA	18.903887728320747	NA	18.92553126013977	NA	19.441821622178967
PP_021730	L-asparaginyl-L-leucine	246.1448835	NA	NA	19.184657458124633	NA	20.00005778501343	20.605756203886177	NA	20.8422743722928
PP_021813	L-Asparaginyl-L-aspartic acid	286.044344	NA	NA	NA	NA	NA	22.370088454490514	NA	NA
PP_022498	L-Threonyl-L-valine	219.133746	19.017732740158813	NA	NA	18.473888040679746	NA	NA	19.30839902859313	NA
PN_008589	Adenosine 2prime,3prime-cyclic phosphate	328.045439	21.891088363699698	21.87907838672871	22.063150481294247	22.23389230006687	22.533847037634295	22.586283181422893	22.753847995839227	22.8051278467574
PN_008925	Cytidine 3prime-phosphate	322.0447099	22.313505852284134	22.175656750808155	22.477245158438855	22.60793641203105	NA	23.328078412055245	NA	23.209954835653306
PN_006270	(15Z)-9,12,13-Trihydroxy-15-octadecenoic acid	329.2334551	20.878514227209763	20.377017723143418	21.290596575365267	22.584370300254573	22.413285997083918	23.40277827488249	22.684242669509377	23.901045486170272
PN_010525	Uridine	243.0621443	21.566084706118154	21.231045938919998	21.338287537644927	22.374424771190657	22.428606552631692	22.755198311398345	22.353939911368762	22.740335404806377
PN_010447	Thymidine	287.0888662	18.0652688618588	17.700175528601353	17.84806661517197	18.72881069951088	18.711542630389932	19.104138010176836	18.627198991254076	19.029505620833348
PN_008781	Azelaic acid	187.0968246	22.437971971156028	22.29846033456897	22.601528248136066	22.657337205471958	22.83769722078014	23.020253110442734	22.62696447519168	23.308405417170377
PN_010519	Uric acid	167.0198795	NA	NA	16.06179307032486	16.219622317071163	NA	16.94761411031805	NA	16.97523223779767
PN_009255	D-Fucose	209.065975	23.086884054128586	23.12272564191069	23.42199827404183	23.656192758815717	23.578298743899612	23.880112978679634	23.462557813026635	23.931356026655482
PN_010879	Dihydrokaempferol	287.0563606	18.953468962228847	18.580015604641826	17.598379687235234	18.977545462113252	17.74089410293774	17.555315591699216	17.244754041464244	18.155114685276992
PN_001200	7-[(6-Deoxyhexopyranosyl)oxy]-5-hydroxy-2-(4-hydroxyphenyl)-4-oxo-4H-chromen-3-yl 2-O-(6-deoxyhexopyranosyl)hexopyranoside	739.2101377	23.495709277329745	23.016648317687956	23.818773974715246	24.124055821321868	24.15770373806995	24.92582518961083	23.671624854310622	24.576752177873864
PN_009955	N-Acetyl-L-methionine	190.0536807	18.31192122798827	18.21714833972697	18.692223855994172	19.05326834185997	18.732674235932482	19.332694386635886	18.883299582183987	19.78267033876737
PN_005616	3-Hydroxy-3-methylpentanedioic acid	161.0445201	21.121527193798904	20.194043822486794	21.294798530753578	NA	21.28356487440679	22.01927817518589	NA	22.1521969392656
PN_010777	Heptanedioic acid	159.0653502	18.245496999801258	18.22949010126639	18.525746365493667	18.62239505948517	18.661858106129078	18.76807768528097	18.395764224027115	18.954378090144047

PN_011938	L-Glutamyl-L-glutamic acid	275.0885956	18.64698755118743	18.02539914095931	18.2723290706833	18.599622321829134	18.87484630473213	19.492737290056766	18.297162572180103	19.61002814663793
PN_008739	alpha-Ketoglutaric acid	145.013157	NA	NA	19.72173337999775	19.660150878526192	NA	19.79605862623256	19.82415233030641	NA
PN_009470	Iminodiacetic acid	132.0291439	19.020242087365236	18.924682045681987	18.787841995324648	19.37602361543811	19.229053485034747	19.509593326363643	19.119308450174035	19.302922234049237
PN_008883	Citric acid	191.0190417	20.449981842007602	20.531468085112056	21.03926648056444	21.053974257190852	21.522965546513205	21.917379000027886	20.37754841060784	21.92273246925365
PN_009536	L-Malic acid	133.0130439	24.811299278725613	25.08519294929786	25.284939467226298	25.26211857990117	25.141194853036048	25.45295414729601	25.339249186984574	25.182181360998555
PN_009931	N-Acetyl-D-phenylalanine	206.0815809	NA	NA	16.08447623745389	16.45341533359415	16.49491806992945	16.775173003008117	16.295697199590126	16.873251842902125
PN_010393	Suberic acid	173.0810271	20.19316316863173	20.15179202089625	20.29571065014252	20.28918813271354	20.428597352477816	20.327359663696203	20.116927704082176	20.477272090647517
PN_011005	Coniferin	387.1299273	20.763944993889766	21.16392909152513	20.66932454382835	20.794314321740465	20.438980638517517	20.4828315855793	20.960386635661514	20.753513024734605
PN_003432	3,5,5-trimethyl-4-[(E)-3-[(2R,3R,4S,5S,6R)-3,4,5-trihydroxy-6-(hydroxymethyl)oxan-2-yl]oxybut-1-enyl]cyclohex-2-en-1-one	415.1977087	18.11080443032397	18.118236459746917	18.328144581938265	18.74030781425232	17.979871725726227	18.057885986661205	17.624595575301253	18.68404315108382
PN_011925	L-Alanyl-L-glutamic acid | L-Glutamyl-L-alanine	217.0827909	17.912684712318224	NA	18.406715725535022	NA	18.89270585531194	18.10412264952948	NA	18.11497250558964
PN_005251	(3E)-4-[(1S)-1-Hydroxy-2,6,6-trimethyl-4-oxo-2-cyclohexen-1-yl]-3-buten-2-yl-beta-D-glucopyranoside	431.1922484	16.508584017342883	16.18338338403176	17.15477856094862	17.464250582819684	16.938614769188014	16.85294396135495	16.497914192754333	17.843933315714605
PN_010963	5-Hydroxyferulic acid	209.0449071	17.573780304048775	17.794872728666974	18.14088966884757	18.358955907823542	17.713983844445433	18.294059779224195	17.78287666575013	18.55643869619706
PN_009589	L-Asparagine	131.045124	21.517242624096596	NA	NA	21.17503353837032	21.613960282410908	NA	20.96519736599155	21.422640218659048
PN_009649	L-Cysteinesulfinic acid	152.0011764	16.104373519625497	NA	NA	17.20064002750416	NA	NA	NA	NA
PN_009937	N-Acetylglutamic acid	188.0557447	20.09470915963816	20.575478310944902	NA	NA	21.043214899739297	NA	NA	NA
PN_010027	N-Formyl-L-aspartic acid	160.0241396	NA	NA	NA	19.439396692342306	NA	18.981558920562016	NA	19.374128887553493
PN_012015	L-Glutamyl-L-valine	245.1140667	17.75687552518317	17.45580078377308	NA	NA	18.186394779298155	NA	NA	NA

Supplementary Table 2. List of the 65 metabolite features detected by LC-MS that showed a statistically different relative abundance between the REs of mock-treated and PCB-18 exposed plants. *Peak_ID*: code for the detected peak. PP, PN: metabolic features detected in polar positive and negative ionization modes, respectively. In the *Compound_name* column, NA stands for not available and indicates unidentified compounds. The remaining columns reported the normalized intensity values for the four biological replicates of REs collected at T2 from the mock treatment (M1, M2, M3, M4) and for the PCB-18 exposed plants (P1, P2, P3, P4).

Peak_ID	Compound_Name	T2M1	T2M2	T2M3	T2M4	T2P1	T2P2	T2P3	T2P4
PP_017474	Scopoletin	22,40548	22,74971	22,33072	21,95557	18,79652	18,71747	18,11246	17,61223
PP_036274	NA	19,97468	19,88211	20,09784	20,24003	21,61341	21,79697	22,1796	21,74798
PP_036639	NA	18,76356	19,13164	18,69681	18,79369	20,80565	20,71235	21,24264	21,29523
PP_037403	NA	20,76659	20,9259	20,89885	21,04463	21,87092	21,86405	21,84342	22,1271
PP_051488	NA	20,89725	20,98597	21,03718	20,95939	20,28458	20,1647	20,34605	20,30947
PP_055290	NA	17,63153	16,98936	17,45458	17,89592	19,66873	20,03977	19,91079	20,42564
PP_030928	NA	22,08319	21,88947	22,03361	22,29175	22,9807	23,15394	23,06306	23,2953
PP_026188	NA	20,63296	20,35831	20,48056	20,81746	21,44181	21,56555	21,52265	21,84306
PP_027809	NA	19,80088	19,77545	20,12759	20,41167	21,25	21,43843	21,54999	21,7452
PP_033414	NA	23,66407	23,69674	23,78821	24,26289	25,04601	25,13504	25,43401	25,43932
PP_033586	NA	19,08807	19,04535	19,23391	19,56465	20,38674	20,7188	20,53143	20,91978
PP_034314	NA	22,87189	22,65213	22,75987	23,0853	23,70102	23,84643	23,78791	24,04693
PP_037084	NA	23,22431	23,601	23,74878	23,88154	24,73494	25,10305	24,90222	25,31841
PP_037201	NA	20,00456	19,92731	20,16668	20,54839	21,34944	21,66274	21,42964	21,83337
PP_037335	NA	23,08553	23,19002	23,10403	23,19704	23,735	23,68402	23,94503	23,84359
PP_037442	NA	18,73767	19,13361	19,23658	19,40452	20,24051	20,59993	20,39983	20,82928
PP_042843	NA	20,73538	20,67473	20,73917	21,4012	22,2765	22,72602	22,32632	22,85757
PP_045961	NA	19,84147	20,2537	20,525	20,36165	21,95505	21,90339	22,37213	21,54407
PP_046320	NA	21,43044	21,50588	21,37039	21,53345	22,03312	21,94165	22,22272	22,11485
PP_055385	NA	23,32564	22,38355	22,53639	22,61071	19,56408	19,42854	19,3944	19,58022
PP_058460	NA	20,63147	20,60371	20,50242	20,67297	20,07077	20,14886	19,9132	20,07208
PP_067423	NA	21,31936	21,49536	21,52127	21,43489	20,91576	20,83748	20,72229	20,99828
PP_031511	NA	20,4597	20,36944	20,39343	20,66472	21,24177	21,42221	21,10674	21,51794
PP_033453	NA	21,53072	20,90404	21,3783	21,71677	22,61346	22,923	23,29588	23,17854
PP_039239	NA	20,53974	20,55077	20,6758	21,30509	22,1576	22,56149	22,17471	22,74414
PP_050413	NA	19,0553	19,68149	19,7304	19,75434	21,05523	21,04028	21,42299	20,90229
PP_036466	NA	19,33223	18,87808	19,5519	19,9236	21,13142	21,44804	21,19897	21,78091
PP_036511	NA	20,57543	20,75929	20,81193	21,21336	21,8264	22,09842	21,96258	22,28682
PP_036313	NA	20,59159	20,75518	20,78442	21,20938	21,82461	22,08664	21,89501	22,22248
PP_036210	NA	20,79896	20,98118	21,10759	20,70023	21,86144	21,71004	21,5481	22,00412
PP_039823	NA	26,69346	26,84803	26,90359	27,31145	27,85811	28,1758	27,96859	28,33214
PP_039995	NA	19,96893	19,41592	20,02637	20,50418	21,64265	21,95097	21,72066	22,33667
PP_040183	NA	22,1539	22,33685	22,37357	22,79242	23,34405	23,62535	23,46079	23,80623

PP_042657	NA	19,46369	19,66811	19,66333	20,10217	20,70085	21,20099	20,75962	21,24081
PP_022099	L-Arginyl-L-valine	17,02724	16,97673	16,87386	17,32677	17,66421	18,04779	17,81245	17,93254
PP_029323	NA	19,82248	20,49387	20,42073	20,20941	21,83468	21,8298	22,42441	21,46259
PP_042040	NA	20,27728	20,52721	20,67641	20,42061	21,17496	21,32942	21,12525	21,06252
PP_050965	NA	19,89378	19,88886	20,13165	20,22222	20,65355	20,9065	20,59119	20,78536
PP_041876	NA	23,88689	24,48791	24,45855	24,26887	25,85156	25,84819	26,4252	25,45852
PP_042264	NA	19,37276	19,99415	19,9712	19,78662	21,3557	21,33223	21,92033	20,94538
PP_045286	NA	21,14337	21,45996	21,53256	21,31978	22,03929	22,19923	22,03306	21,92773
PP_049587	NA	21,27324	21,33122	21,18644	21,35655	21,80169	21,73627	22,05713	21,90794
PP_066692	NA	18,93599	18,29485	18,29982	18,88552	19,7751	20,0507	20,12995	20,67918
PP_028990	NA	20,68537	20,72957	20,53259	20,40033	21,60902	21,34437	21,13297	21,28259
PP_043865	NA	21,02394	20,87324	20,71264	20,68824	20,17163	20,23463	20,06699	20,39742
PP_030754	NA	25,59941	25,1026	25,53693	25,71588	26,328	26,4903	26,67543	26,6334
PP_016782	N-(2-Hydroxyethyl)-beta-alanine	19,60801	19,739	19,64165	19,62358	19,21726	19,14683	18,98386	19,2676
PP_031075	NA	21,13269	20,61586	21,10143	21,20988	21,84985	22,01873	22,18158	22,14667
PP_033780	NA	22,44156	22,73858	22,66497	23,1565	23,51796	23,90975	24,02219	23,92027
PP_031078	NA	19,09414	19,42857	19,19086	19,38376	20,37094	20,37488	19,88088	20,05649
PP_039077	NA	21,67966	21,69025	21,77962	21,64848	23,38066	23,47852	24,04673	23,25721
PP_030654	NA	19,83433	20,17459	20,09283	20,57339	20,94296	21,32222	21,42589	21,37652
PP_033321	NA	21,33897	21,48869	21,30025	21,93556	22,25779	22,56647	22,81594	22,62292
PP_040170	NA	20,86781	21,5157	21,52289	21,77496	22,97809	23,08623	22,62252	22,90118
PP_048016	NA	22,72899	21,84702	22,39435	22,02355	23,5866	23,69198	24,14606	23,30691
PP_042043	NA	22,50229	21,84101	22,28137	23,18204	24,04611	24,68344	24,23488	24,78113
PP_053057	NA	19,93724	20,20368	20,05717	20,26397	20,65733	20,70761	21,1145	20,84528
PP_036696	NA	24,76032	24,94225	24,71269	25,36631	25,68092	25,96957	26,24278	26,01343
PP_042613	NA	19,34012	19,64276	19,45697	19,79595	19,02894	18,96481	18,67008	18,74692
PP_045431	NA	22,4803	21,79225	22,2023	23,13631	24,00343	24,59661	24,20509	24,73485
PP_016186	Hypoxanthine	18,11037	17,672	17,03029	18,12612	19,23426	19,17162	19,57336	20,1741
PP_022339	L-Seryl-L-phenylalanine	18,89562	18,55654	18,70878	19,12457	19,37742	19,74125	19,69943	19,82436
PN_014172	NA	19,46	19,97	20,21	19,91	23,66	23,86	24,23	23,98
PN_014864	NA	25,68	25,47	25,50	25,37	24,73	24,75	24,49	24,45
PN_030544	NA	23,28	23,07	23,12	23,03	22,30	22,22	22,02	22,01

Supplementary Table 3. PERMANOVA analysis on the effect of treatment (mock or PCB-18) on the metabolite fingerprint detected by positive and negative mode by LC-MS. Df: degrees of freedom; MS: mean sum of squares; Pseudo-F: F value by permutation; P: p value.

**Positive Mode**	df	MS	Pseudo-F	P
Treatment	1	1028,6	3,764	0,0286
**Negative Mode**	df	MS	Pseudo-F	P
Treatment	1	360,92	2,8685	0,0283

Supplementary Table 4. Values of *Acinetobacter* P320 and *Pseudomonas* JAB1 doubling time when exposed to increasing concentrations (250 µM to 2 mM) of scopoletin. The doubling time was indicated as hours:minutes. The plant secondary metabolite was dissolved in 80% methanol, indicated here as MetOH. Statistical analysis was performed using the Mann-Whitney test, by comparing the scopoletin treatment with the mock solvent control. ***: $p \leq 0.001$; ns: non-significant ($p > 0.05$).

**Bacterium**	**Treatment**	**Concentration**	**Doubling time**	**Statistical analysis**
Acinetobacter P320	MetOH	80%	3:14 ± 0:33	
	Scopoletin	250 µM	3:37 ± 0:46	ns
		500 µM	4:12 ± 0:45	ns
		1 mM	4:19 ± 0:27	***
		2 mM	5:32 ± 0:23	***
Pseudomonas JAB1	MetOH	80%	2:36 ± 0:24	
	Scopoletin	250 µM	2:36 ± 0:34	ns
		500 µM	2:47 ± 0:37	ns
		1 mM	3:25 ± 0:20	***
		2 mM	3:30 ± 0:37	ns

SUPPLEMENTARY FIGURES

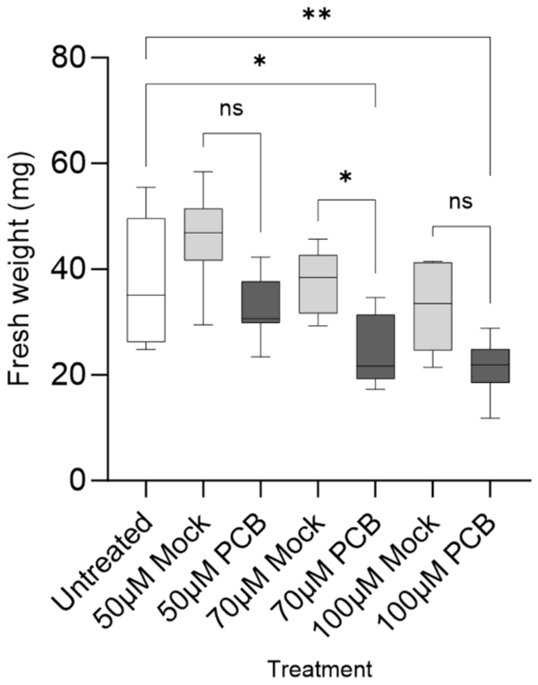

Supplementary Figure 1. *Arabidopsis thaliana* sensitivity to PCB-18 phytotoxic effects. This assay was fundamental to establish the concentration of PCB-18 to be used to trigger *in vitro* PCB-18 stress. Seven days post-germination in plate, *Arabidopsis* plantlets were transferred in a 24-well plate containing ½ MS liquid medium supplemented with increasing concentrations of PCB-18 or an equal volume of acetone, the solvent in which the pollutant was resuspended. The untreated sample corresponds to plants that were not exposed neither to acetone nor to PCB-18. Each well contained 3 plantlets, three/four technical replicates were used and the experiment was repeated twice. The plantlets in each well were pooled and weighed by using a precision scale. Acetone application in mock treated samples did not interfere with plant growth, showing similar plant fresh weight values if compared with untreated plantlets. It was observed that at 50 µM, PCB-18 application caused a 28.4 ± 1.8 % decrease compared to the mock treatment. At 70 and 100 µM concentrations, a stronger PCB-18-triggered phytotoxic effect was observed compared to 50 µM, although the percentage decrease was quite similar and corresponded to 35.8 ± 4.5 % and 34.7 ± 11 % respectively. Considering a lower sample variability, 70 µM concentration was selected to identify the PCB-triggered ‘cry-for-help’ exudation pattern.

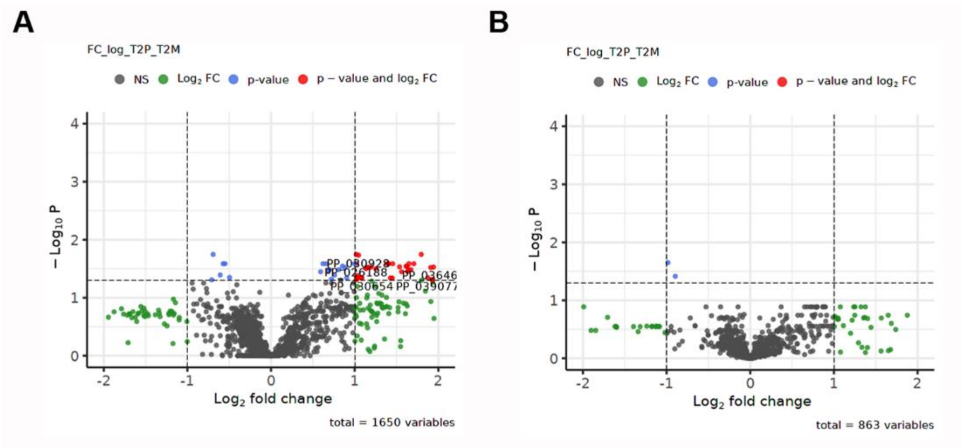

Supplementary Figure 2. Volcano plots of the untargeted metabolomic analysis to investigate PCB-triggered changes in *Arabidopsis* root exudation pattern. (A), (B): volcano plots of the detected metabolite features obtained by LC-MS in the positive polarity and in the negative polarity mode, respectively. These data represent all metabolite features with a fold change that was >$\log_2|1|$ and for which the p-values of the two-tailed t-test was corrected using the Benjamini-Hochberg (BH) method.

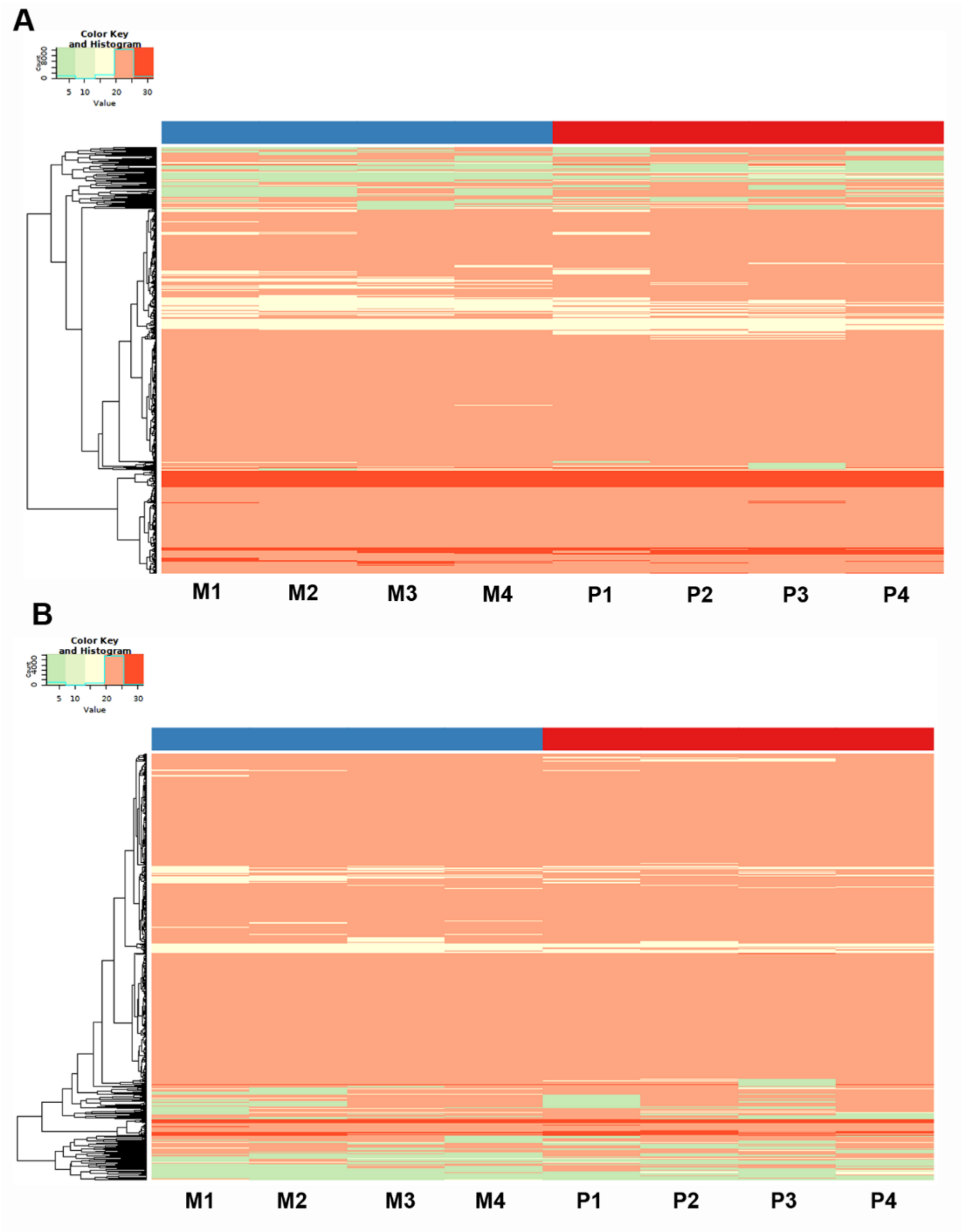

Supplementary Figure 3. Heat-map of the metabolite features detected by untargeted metabolomics through LC-MS analysis. (A), (B) Heatmaps of the hydrophilic metabolites detected through LC-MS in positive and negative polarity mode, respectively. The graphs represent the root exudates released by plantlets exposed for 2 days (T2) challenged with 70 µM PCB-18 (P, in red in the graph) or mock-inoculated with an equal amount of acetone (M, in blue in the graph).

Supplementary Figure 4. Flavonoids did not show statistically relevant variations in the metabolomic dataset. In the graphs, the boxplots show the relative intensity of the flavonoid metabolites detected in the analysis, expressed as log_2 of the normalized intensity.

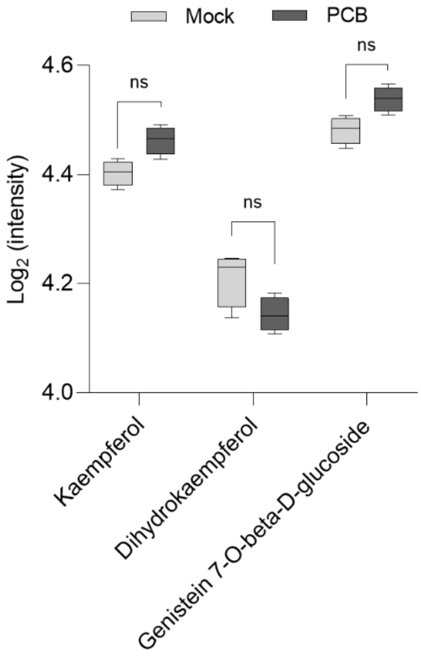

Supplementary Figure 5. Microscopy analysis of the root colonization profile of the fluorescent-labeled PCB-degrading strains used in the present study. Epifluorescence microscopy analysis of the colonization pattern of *gfp*-labelled *Acinetobacter* P320, *mScarlet*-labelled LB400 strain and *mScarlet*-labelled JAB1 strain on the root system of WT *Arabidopsis* plantlets at 12 days post germination.

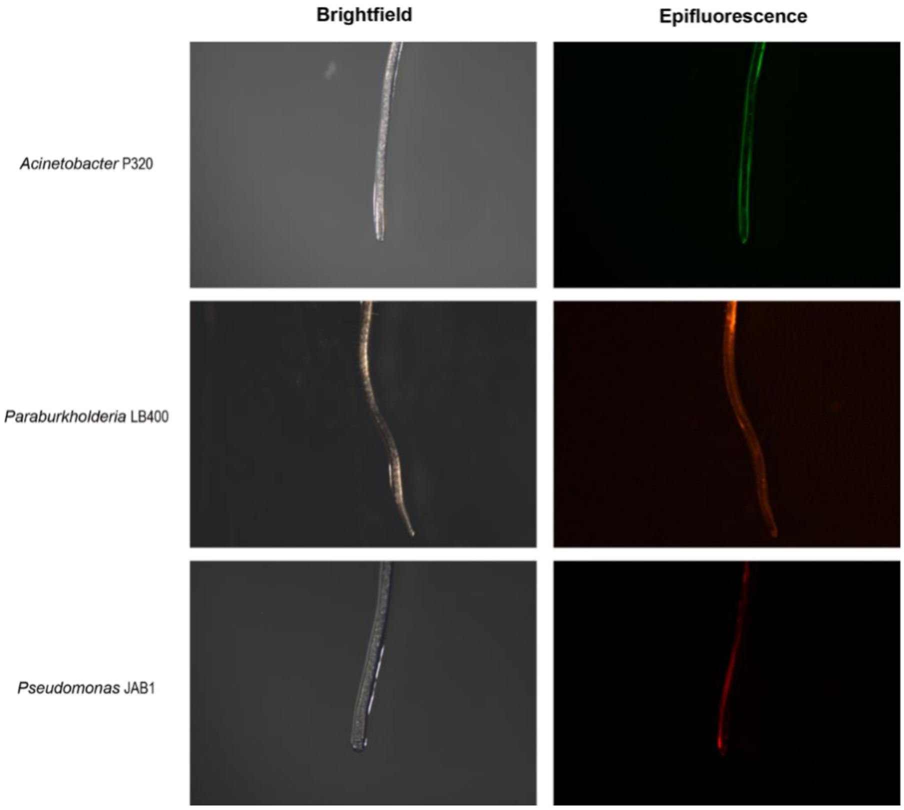

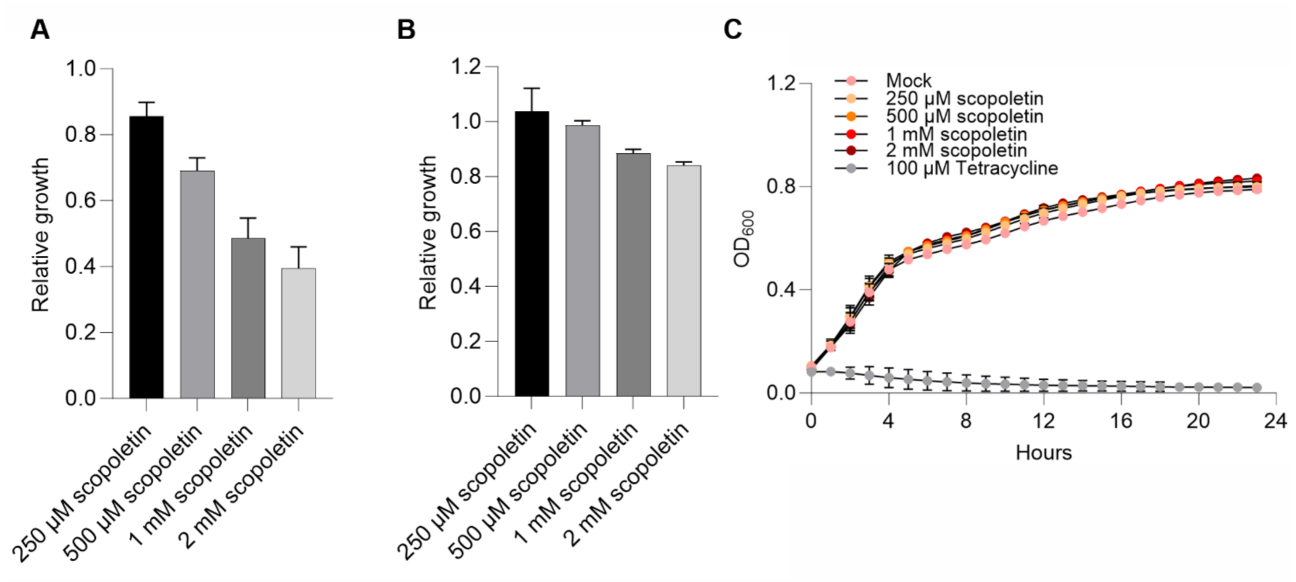

Supplementary Figure 6. Scopoletin affected the growth of the selected PCB-degrading strains. (A) and **(B)** *Acinetobacter* P320 and *Pseudomonas* JAB1 relative growth, respectively, at increasing concentrations of scopoletin. The bars represent the ratio between the OD_{600} value of the culture in presence of scopoletin and the OD_{600} of the control culture grown in presence of the solvent used to resuspend the plant secondary metabolite. **(C)** *P. xenovorans* LB400 growth is not affected by scopoletin supplements to the medium. In the assay, 100 µM tetracycline was used as a positive control for growth inhibition.

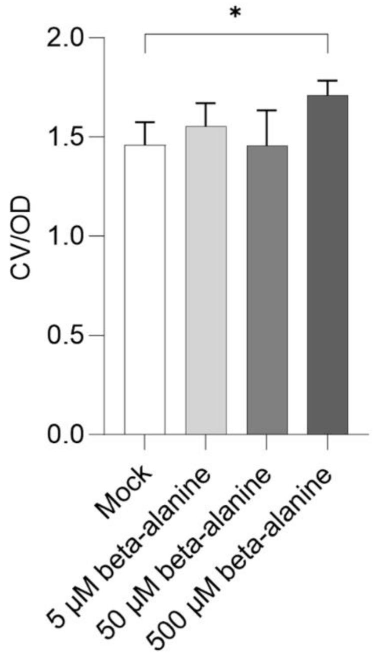

Supplementary Figure 7. N-hydroxyethyl-beta-alanine positively affected strain LB400 ability to form a biofilm. Biofilm formation ability of strain LB400 was represented as the ratio between the crystal violet OD (CV) of the stained biofilm and the optical density of the culture at 600 nm (OD). Statistical analysis was performed using the Mann-Whitney test (n=3), by comparing the β-alanine treatment with the mock solvent control. *: $p \leq 0.05$.

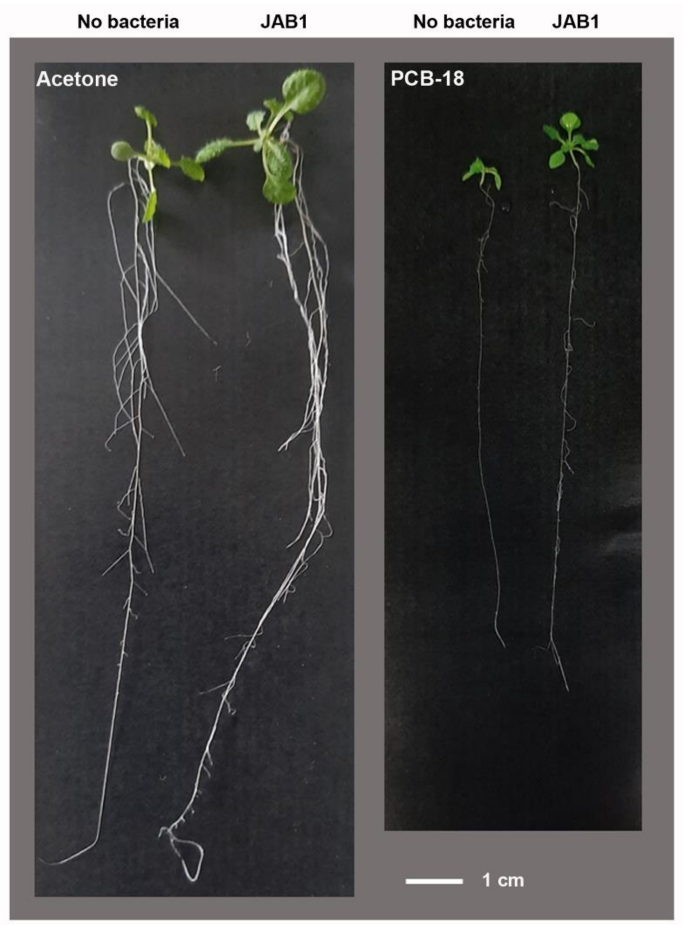

Supplementary Figure 8. ***Pseudomonas*** **JAB1 is a plant growth promoting bacterium and is able to stimulate** ***Arabidopsis*** **growth under PCB stress.** Representative images of *Arabidopsis* WT plantlets colonized by strain JAB1 or sterile (no bacteria) grown for 14 days under mock treatment (acetone) or exposed to 20 µM PCB-18.

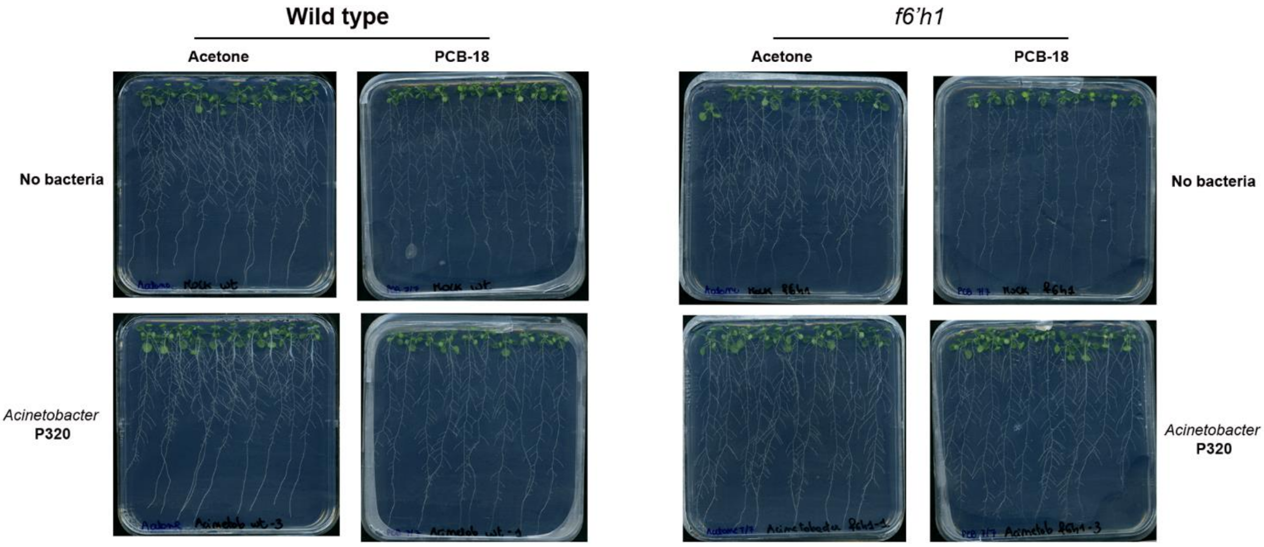

Supplementary Figure 9. *Acinetobacter* P320 was able to boost plant growth under control condition and under PCB-18 stress. Representative images of *Acinetobacter* P320-induced growth promotion in *Arabidopsis* plantlets in WT and *f6'h1* line under mock treatment (acetone) and PCB-18 induced stress. The brightness of the images was enhanced for all the displayed samples (contrast value 84 by using Photoshop CS5 Extended v.12) to improve the visibility of the root system.

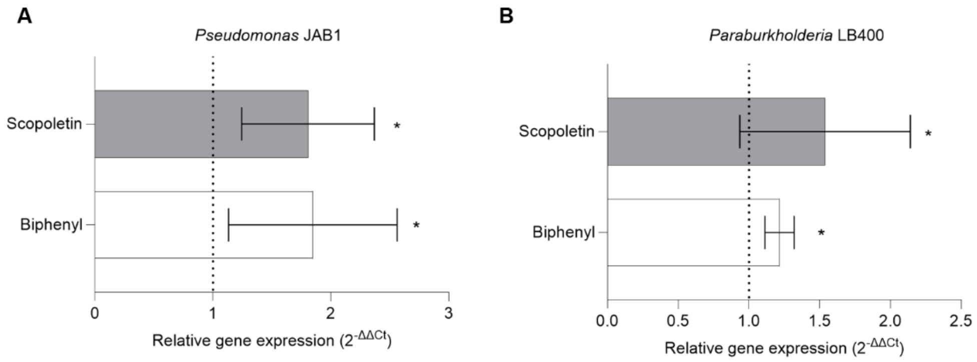

Supplementary Figure 10. Scopoletin induced the *bphA* expression in PCB degrading bacteria. (A) *bph* induction *Pseudomonas* JAB1 and (B) in *Paraburkholderia* LB400 by scopoletin. The relative gene expression of *bphA* gene was quantified via RT-qPCR, using the *infB* gene as housekeeping gene for *Pseudomonas* JAB1 and the 16S rRNA gene for *Paraburkholderia* LB400. The results of the relative expression of *bphA* transcripts are expressed as ΔΔCt, indicating the average fold change over the non-induced control. The black line (relative gene expression value = 1) represents the baseline of gene expression in the non-induced control. Biphenyl was used as positive control. Error bars represent the standard deviation of 3 independent experiments. Biphenyl induction in the experiments with strain LB400 was performed with two biological replicates. Statistical analysis was performed using the Mann-Whitney test (n=2-3), *: $p \leq 0.05$.

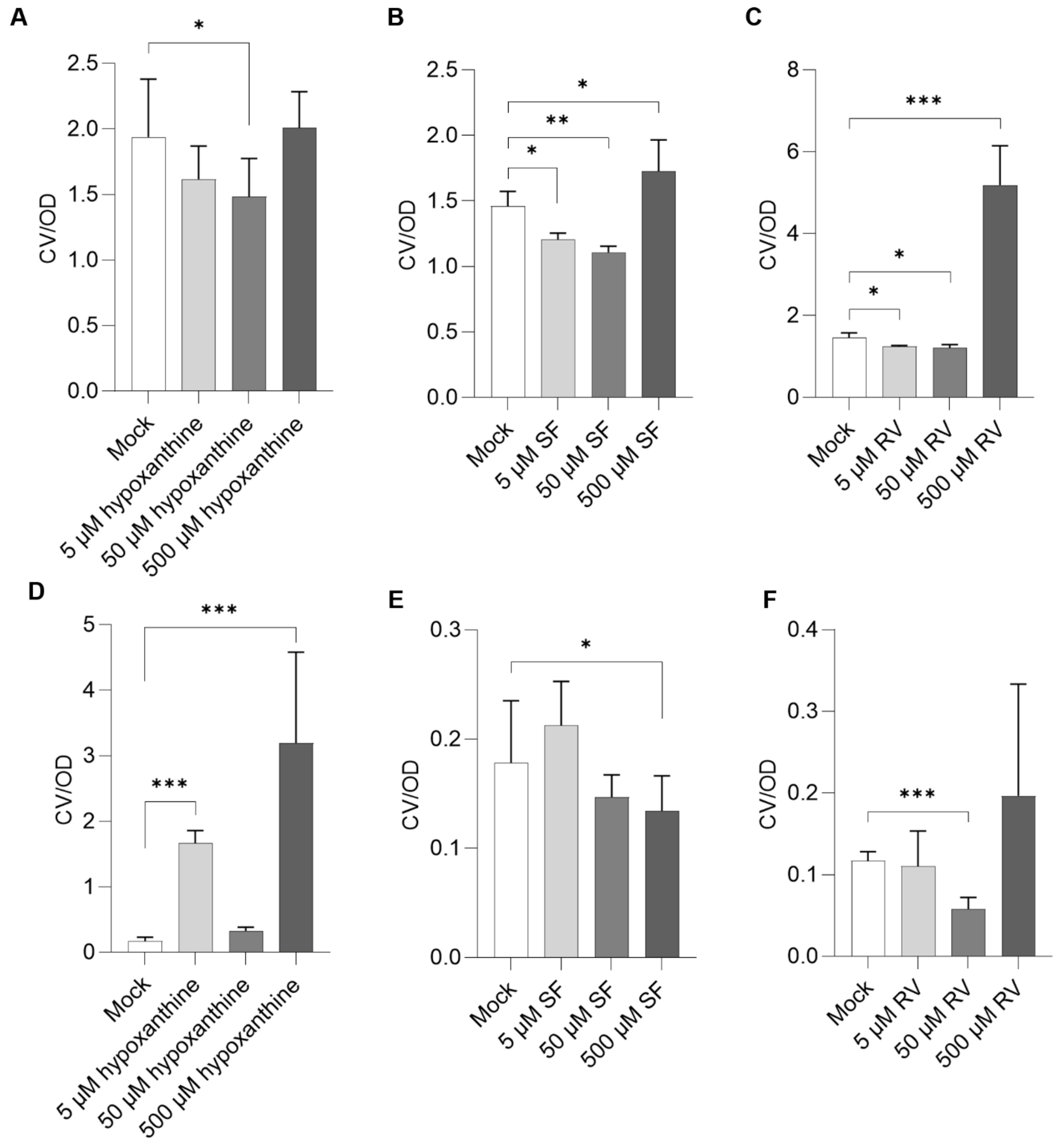

Supplementary Figure 11. Effects of hypoxanthine, seryl-L-phenylalanine (SF) and L-arginyl-L-valine (RV) on biofilm formation ability of *Paraburkholderia* LB400 and *Pseudomonas* JAB1. (A), **(B)** and **(C)** biofilm formation ability in *Paraburkholderia* LB400 at increasing concentrations of hypoxanthine, SF and RV dipeptides, respectively. **(D)**, **(E)** and **(F)** biofilm formation ability in *Pseudomonas* JAB1 at increasing concentrations of hypoxanthine, SF and RV dipeptides, respectively. Biofilm formation ability of the strains was represented as the ratio between the crystal violet OD (CV) of the stained biofilm and the optical density of the culture at 600 nm (OD). Statistical analysis was performed using the Mann-Whitney test (n=3), by comparing the compound treatment with the mock solvent control. *: $p \leq 0.05$; **: $p \leq 0.01$, ***: $p \leq 0.001$.
